# Characterization of Korean Colorectal Cancer Reveals Novel Driver Gene and Clinically Relevant Mutations

**DOI:** 10.1002/mco2.70584

**Published:** 2026-01-08

**Authors:** Junho Kang, Dong Min Lim, Young‐Joon Kim, Hyeran Shim, Tae‐You Kim, Kyu Joo Park, Sung‐Bum Kang, Chang Sik Yu, Jong Lyul Lee, Yeuni Yu, Hansong Lee, Eun Jung Kwon, Hyo Min Kim, Seongik Mun, Donghee Kwak, Hae Seul Lee, Hye Jin Heo, Eun Kyoung Kim, Seung Eun Baek, Jong‐Wook Park, Sung Uk Bae, Taeg Kyu Kwon, Dongjun Lee, Kihun Kim, Chang‐Kyu Oh, Dai Sik Ko, Sunghwan Cho, Hae Ryoun Park, Shin Kim, Yun Hak Kim

**Affiliations:** ^1^ Department of Research Keimyung University Dongsan Medical Center Daegu Republic of Korea; ^2^ Medical Research Institute Pusan National University Yangsan Republic of Korea; ^3^ Department of Biochemistry, College of Life Science and Biotechnology Yonsei University Seoul Republic of Korea; ^4^ Department of Internal Medicine Seoul National University College of Medicine Seoul National University Hospital Seoul Republic of Korea; ^5^ Department of Surgery, Seoul National University Hospital Seoul National University College of Medicine Seoul Republic of Korea; ^6^ Department of Surgery, Seoul National University Bundang Hospital Seoul National University College of Medicine Seongnam Republic of Korea; ^7^ Department of Surgery, Division of Colon and Rectal Surgery University of Ulsan College of Medicine and Asan Medical Center Seoul Republic of Korea; ^8^ Department of Convergence Medicine, School of Medicine Pusan National University Yangsan Republic of Korea; ^9^ Department of anatomy, School of Medicine Pusan National University Yangsan Republic of Korea; ^10^ Department of Immunology, School of Medicine Keimyung University Daegu Republic of Korea; ^11^ Institute of Medical Science Keimyung University Daegu Republic of Korea; ^12^ Department of Surgery Keimyung University Dongsan Medical Center Daegu Republic of Korea; ^13^ Center for Forensic Pharmaceutical Science Keimyung University Daegu Republic of Korea; ^14^ Department of Occupational and Environmental Medicine Pusan National University Yangsan Hospital Yangsan Republic of Korea; ^15^ Department of Biochemistry, School of Medicine Pusan National University Yangsan Republic of Korea; ^16^ Division of Vascular Surgery, Department of General Surgery Gachon University Gil Medical Center Incheon Republic of Korea; ^17^ Department of Surgery Pusan National University Yangsan Hospital Yangsan Republic of Korea; ^18^ Department of Oral Pathology, School of Dentistry Pusan National University Yangsan Republic of Korea; ^19^ Department of Biomedical Informatics School of Medicine Pusan National University Yangsan Republic of Korea

**Keywords:** colorectal cancer, microsatellite instability, mutational hotspots, tumor mutational burden, whole‐genome sequencing

## Abstract

Colorectal cancer (CRC) ranks as the third leading cause of cancer‐related deaths worldwide, characterized by genomic heterogeneity arising from ethnic and interindividual differences. Producing region‐specific data to characterize ethnic‐specific somatic mutations is essential for advancing CRC research. Additionally, accurate somatic mutation detection requires paired tissue analyses to account for interindividual diversity. This study aims to highlight the importance of ethnic diversity in shaping CRC's genomic landscape and emphasize the necessity for region‐specific data to refine diagnostic and therapeutic approaches. This study emphasizes the need for region‐specific data by analyzing an unprecedented 197 paired samples from the Korean CRC cohort through whole‐genome sequencing. We identified 78 potential driver genes. Notably, *CBWD5*, *LRRIQ3*, *TRIM64B*, *SPINK5*, and *ZNRF2* were linked to recurrence, presenting potential therapeutic targets. Our analysis revealed 30 mutational hotspots, with significant variants in *KRAS* (25%, G12A, G12D, G12V), *MAP1A* (12%, V2300G), and *TP53* (8%, R175H). We identified a significant co‐occurrence between *KRAS* 12 mutation and *PIK3CA* 545 mutation. Our findings demonstrate potential driver genes and mutational hotspots associated with CRC patient, characterizing the mutational landscape related to clinical characteristics. Significantly advancing our understanding of CRC's heterogeneous nature, this study lays a solid foundation for devising more efficacious management strategies.

## Graphical abstract

This study utilizes whole‐genome sequencing of 197 paired samples to conduct mutational profiling of colorectal cancer (CRC) in Korean patients, identifying novel driver genes associated with recurrence. The research provides insights into oncogenic pathways and highlights clinically significant variants, emphasizing the importance of ethnic diversity in CRC patient stratification.

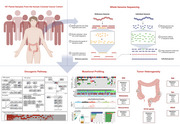

## 1 Introduction

Colorectal cancer (CRC) is the third most common cancer and the third leading cause of cancer deaths worldwide [1]. Despite significant advancements in therapeutic strategies, including targeted agents and immunotherapies, a comprehensive understanding of the mechanisms underlying colorectal carcinogenesis and therapeutic resistance remains insufficient. The 5‐year survival rate drops markedly in patients with advanced‐stage disease, primarily due to tumor heterogeneity, late detection, and limited treatment response [2]. Recent progress in both primary and adjuvant therapies has led to gradual improvements in CRC survival outcomes. In clinical settings, the fundamental goal of treatment is complete removal of the primary tumor and metastatic lesions, which typically necessitates surgical resection [3]. However, despite the implementation of large‐scale screening programs intended to lower CRC incidence and detect early‐stage disease, approximately 25% of patients are still diagnosed with distant metastases, and an additional 20% of initially localized cases eventually develop metachronous metastases [4, 5, 6, 7]. These conditions significantly hinder curative surgical management and contribute to high mortality rates. For patients with unresectable tumors or those who are not candidates for surgery, the treatment objective shifts toward maximal tumor shrinkage and suppression of disease progression. In such cases, radiotherapy and systemic chemotherapy remain the primary therapeutic modalities. Moreover, in selected patients, these treatments may be applied in a neoadjuvant or adjuvant setting to facilitate surgical resection and reduce the likelihood of recurrence by eliminating micrometastatic disease or stabilizing the tumor burden [8, 9, 10, 11].

Given the considerable heterogeneity among CRC cases and the variability in treatment responses, there is a critical need for improved patient stratification and personalized therapeutic strategies. The continuous advancement of next‐generation sequencing technologies has propelled the era of precision oncology [12]. For example, in non‐small cell lung cancer, whole‐exome sequencing (WES) has enabled the identification of clinically actionable gene amplifications such as *ERBB2* and *MET* [13]. Similarly, comprehensive genomic sequencing studies in high‐grade serous ovarian and prostate cancers have elucidated key resistance mechanisms—including *BRCA1/2* reversion mutations and *MDR1* overexpression in ovarian cancer, as well as a long tail of statistically significant mutations in prostate cancer—underscoring the utility of large‐scale genomic profiling in uncovering therapeutic vulnerabilities and enhancing my understanding of tumor evolution [14, 15].

Among these advancements, identifying somatic mutations in CRC holds significant potential for characterizing individual tumor profiles and informing diagnostic and therapeutic strategies [2]. However, previous genomic studies of Korean CRC have been conducted on WES cohorts with low sample sizes, which has constrained comprehensive variant detection and the accurate characterization of somatic mutations in this population [16, 17]. Precision oncology—which depends on accurate diagnosis and personalized treatment—is enabled by the reliable identification of somatic mutation signatures through tumor–normal paired sequencing, which helps reduce false‐positive somatic calls [18]. Therefore, it is essential to recognize two critical aspects within the field of CRC genome research. First, the existence of interethnic somatic mutation disparities, which extend beyond CRC to encompass various cancer types, emphasizes the necessity for studies on interethnic diversity. Second, the interindividual diversity among human populations emphasizes the imperative for paired tissue analyses to enhance the reliability of somatic mutation detection [19, 20, 21]. In the pursuit of precision oncology, it is imperative to address racial and ethnic disparities. Recent studies have highlighted molecular discrepancies in early‐onset CRC (EOCRC) across racial/ethnic and sex lines. Consequently, comprehending the disparities among racial and ethnic groups is invaluable, particularly in the context of precision medicine research. This highlights the importance of understanding population cohorts for collecting biological and biomarker data applicable to broader cancer populations [22, 23, 24, 25].

Driver mutations are causally involved in the tumorigenic process and are positively selected during tumorigenesis. However, a single gene defect does not “cause” cancer. It is becoming increasingly clear that pathways govern the tumorigenic process. Therefore, mutations in any one of several genes in a single pathway can result in an equivalent increase in net cell proliferation. Consequently, genes within a particular pathway may mutate more frequently than predicted by chance [26, 27, 28].

We analyzed 197 paired whole‐genome sequencing (WGS) samples from a Korean CRC cohort to identify somatic mutations confidently and explore interindividual differences. We aimed to uncover novel driver genes and mutational hotspots linked to CRC patient outcomes and characterize the mutational landscape related to key clinical features such as age, sex, tumor location, microsatellite instability (MSI), recurrence, and metastasis. Our findings elucidate specific pathways affected by these mutations, offering insights into oncogenic pathways and unique mutations in CRC across ethnic and individual diversity.

## 2 Results

### 2.1 Driver Genes and Hotspot Variants Are Associated With CRC Relapse

Tumor prognosis and treatment efficacy prediction rely significantly on the identification of driver genes and mutational hotspots. We identified a total of 78 driver genes, which include well‐known genes such as *TP53*, *KRAS*, and *APC*, previously established as driver gene in CRC (Table S1). Subsequently, we identified recurrent variants at specific loci within genes, defining them as hotspots. Our findings revealed that mutations at the *KRAS* 12 codon were observed in 49 out of 197 cases (29%, including G12D‐29pts, G12V‐16pts, G12A‐4pts), followed by *MAP1A* mutations in 24 out of 197 cases (12%, V2300G), and *TP53* mutations in 16 out of 197 cases (8%, R175H), showing high frequencies at single positions (Table S2). We evaluated selected driver genes for their association with outcomes using a Cox proportional hazard model to investigate the impact of various variables on disease‐free survival (DFS). Specifically, we examined the influence of the following factors: *CBWD5* (HR: 4.978; CI: 1.21–20.56; *p* value: 0.0265*), *LRRIQ3* (HR: 4.044; CI: 0.56–29.023; *p* value: 0.1647), *TRIM64B* (HR: 46.200; CI: 4.93–433.30; *p* value: 0.0008***), *SPINK5* (HR: 13.0810; CI: 2.43‐70.30; *p* value: 0.0027*), *ZNRF2* (HR: 70.760; CI: 4.98–1004.67; *p* value: 0.0017**), age, sex, disease stage, hypermutation status, and MSI status. By integrating these factors into our analysis, our objective was to clarify their individual and collective associations with patient DFS in our cohort (Tables 1 and S3).

**TABLE 1:** Cox proportional hazards analysis of prognostic driver genes in patients with CRC.

Variable	HR^1^	95% CI^2^	*p* Value
*CBWD5*	4.978	1.21–20.56	0.0265^*^
*LRRIQ3*	4.044	0.56–29.023	0.1647
*TRIM64B*	46.200	4.93–433.30	0.0008^***^
*SPINK5*	13.080	2.43–70.30	0.0027^**^
*ZNRF2*	70.760	4.98–1004.67	0.0017^**^
Age	0.983	0.94–1.03	0.4935
Sex			
Female	Reference		
Male	0.371	0.12–1.17	0.0914
Disease stage			
I	Reference		
II	3.00E+07	0	0.9992
III	1.44E+08	0	0.9991
IV	1.090	0	1.0000
Hypermutation			
Hyper	Reference		
Non‐hyper	8.000	0.32–197.35	0.2034
MSI status			
MSI high	Reference		
MSI low	4.13E−09	0	0.9986
MSS	0.340	0.015–7.69	0.4972
Unknown	2.07E−09	0	0.9996

Concordance = 0.83, likelihood ratio test *p* = 0.003, Wald test *p* = 0.04, Log‐rank test *p* = 0.0001.

^1^HR: adjusted hazard ratio, estimated using the Cox proportional hazards model.

^2^CI: confidence interval.

### 2.2 Somatic Mutation Landscape of CRC

**FIGURE 1:**
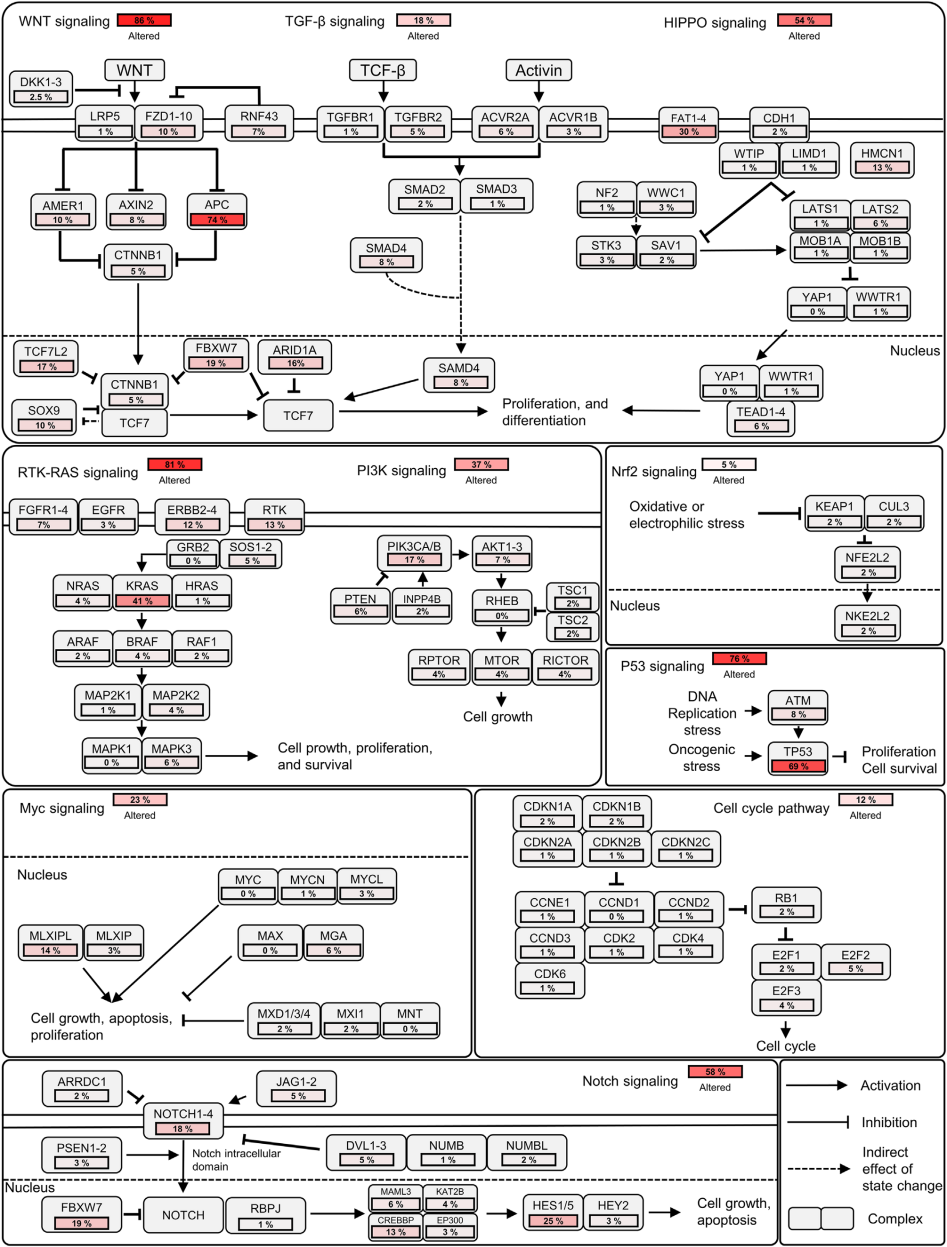
Alterations in 10 oncogenic pathways of CRC in 197 Koreans. Each pathway diagram shows the proportion of patients harboring mutations in the indicated genes, with red shading representing higher mutation frequencies. Percentages beside each pathway denote the proportion of patients with at least one mutation in that pathway. Solid and dotted lines indicate the cell and nuclear membranes, respectively; arrows indicate activation, bars indicate inhibition, and dotted arrows denote indirect regulatory effects.

**FIGURE 2:**
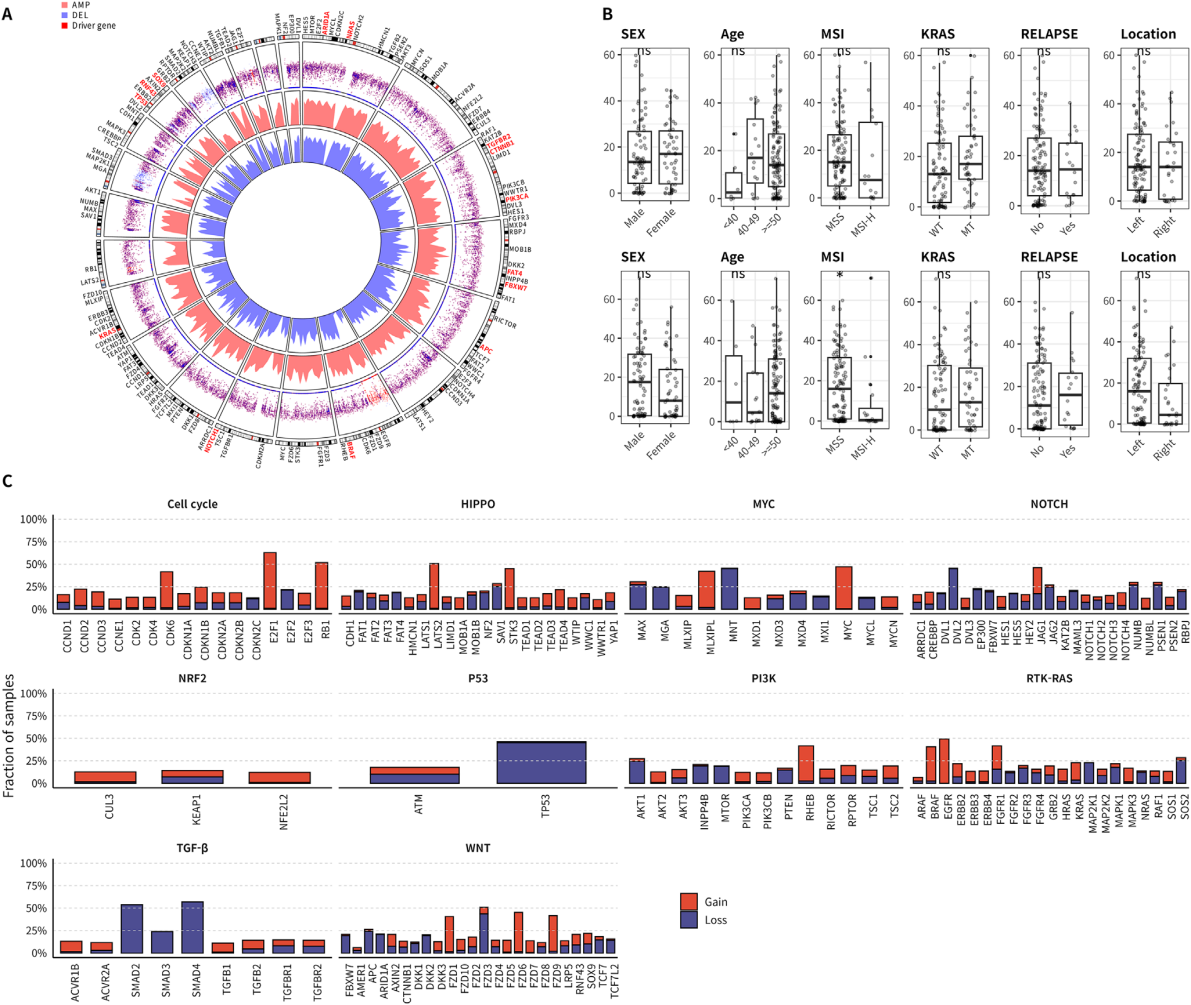
Somatic CNVs in Korean CRC patients. (A) Circos plot showing genome‐wide CNV patterns. The outermost ring represents chromosomes; red and blue dots indicate amplification and deletion degrees, respectively. Inner heatmaps display amplification in red and deletion in blue, with red‐labeled genes showing the highest CNV levels. (B) Boxplots showing CNV frequencies across clinical variables: sex, age, MSI status, KRAS mutation, relapse status, and tumor location. Gains (upper, red) and losses (lower, blue) are indicated. (C) CNV distribution across 10 oncogenic pathways. Red and blue bars represent gain and loss frequencies in key genes of major signaling pathways.

We characterized the somatic variants in Korean CRC patients. Single nucleotide variants (SNVs) were the most prevalent mutation type, followed by small deletions (DELs) and insertions (INSs). C>T transitions were the most frequent base substitution, and missense mutations constituted the predominant functional class. Frequently mutated genes included *TTN*, *APC*, and *PABPC1* (Figure S1A–E). We then compared the tumor mutational burden (TMB) distribution between the Korean CRC cohort and 33 The Cancer Genome Atlas (TCGA) cancer types. Cancer types were ordered by increasing median TMB. The TMB levels in Korean CRC were comparable to those observed in TCGA‐CRC (COAD and READ), placing Korean CRC among the cancer types with relatively high TMB (Figure S1F). For assessing oncogenic pathway alterations, we focused on 10 pathways with known frequent genetic changes. Within these, we depicted genes with higher alteration frequencies in our cohort of 197 Korean CRC patients and calculated the proportion with at least one alteration in these genes. High alteration rates were observed in the WNT (86%), RTK–RAS (81%), P53 (76%), NOTCH (58%), HIPPO (54%), and PI3K–AKT (37%) pathways. The remaining pathways had lower alteration rates: MYC (23%), TGF‐beta (18%), cell cycle (12%), and NRF2 (5%). Within the 10 oncogenic pathways in our cohort, key genes with significant mutations were *APC* (74%), *TP53* (69%), *KRAS* (41%), and *FBXW7* (19%), in that order (Figure 1). To investigate ethnic‐specific differences in oncogenic signaling, we compared the mutational profiles of oncogenic pathways between the Korean CRC cohort and the TCGA‐CRC cohort [29]. Representative oncogenes and tumor suppressor genes within each pathway were assessed to enable an integrative interpretation of interethnic variation. As shown in Figure S1G, the Korean CRC cohort exhibited significantly higher mutation frequencies in the HIPPO (*p* value: <0.0001****), Notch (*p* value: <0.0001****), and Nrf2 (*p* value: 0.0044**) pathways compared with TCGA‐CRC. In contrast, the TCGA‐CRC cohort showed significantly higher mutation frequencies in the cell cycle (*p* value: <0.0001****) and TGF‐β (*p* value: 0.0035**) signaling pathways. No significant differences were observed between the two cohorts in the Myc (*p* value: 1.0000), PI3K (*p* value: 0.3960), RTK–RAS (*p* value: 0.0615), P53 (*p* value: 0.1270), and WNT (*p* value: 0.0598) pathways. We next examined gene‐level alterations to identify population‐specific differences and to evaluate the mutational status of established CRC driver genes (Figure S1H). Among genes with notable inter‐cohort differences, *CDKN2A*, a tumor suppressor gene involved in the cell cycle pathway, was mutated in 32% of TCGA‐CRC cases but was infrequently altered in the Korean CRC cohort (1%). In contrast, *RPTOR*, an oncogenic component of the PI3K–mTOR pathway, exhibited a higher mutation frequency in Korean CRC (13%) compared with TCGA‐CRC (1%), suggesting potential cohort‐specific activation of this signaling axis. Within the HIPPO pathway, *FAT1*, a tumor suppressor gene involved in maintaining epithelial architecture and suppressing tumor progression, was more frequently mutated in TCGA‐CRC (30%) than in Korean CRC (14%). In the MYC regulatory network, *MGA*, a negative regulator of MYC transcriptional activity, showed a higher alteration frequency in TCGA‐CRC (18%) compared with Korean CRC (6%). With respect to well‐established driver genes, *TP53* and *APC*—two of the most frequently mutated tumor suppressors in CRC—exhibited consistently high mutation frequencies in both cohorts, highlighting their conserved roles in CRC pathogenesis regardless of ethnicity. To assess large‐scale genetic variations, we performed copy number variation (CNV) analysis on 197 Korean CRC patients. CNV analysis revealed significant amplifications in key oncogenes, including *KRAS*, *MYC*, and *PIK3CA*, which are known to play crucial roles in CRC progression (Figure 2A). These findings support the involvement of *KRAS*, *MYC*, and *PIK3CA* in tumorigenesis, particularly through pathway‐specific amplifications that drive tumor growth and survival. We then examined the relationship between CNVs and clinical variables. Our results showed that DELs were significantly more frequent in the microsatellite stable (MSS) group compared with the MSI‐high (MSI‐H) group, with CNVs observed only in the MSI status, indicating that CNVs were specifically associated with MSI status (Figure 2B). In the context of oncogenic pathways, we observed significant CNVs in several pathways. Notably, WNT, RTK–RAS, and PI3K–AKT pathways exhibited high frequencies of gain mutations, while TGF‐β and cell cycle pathways showed relatively lower alteration rates (Figure 2C). These findings suggest that pathway‐specific CNVs may contribute to the molecular heterogeneity of CRC and play a significant role in its progression. Among the most frequently altered genes in these pathways, *TP53*, *APC*, and *FBXW7* exhibited significant DEL frequencies, highlighting their roles as key tumor suppressors in CRC.

### 2.3 Hypermutation Characterizes a Unique Mutational and Pathway Profile in CRC

**FIGURE 3:**
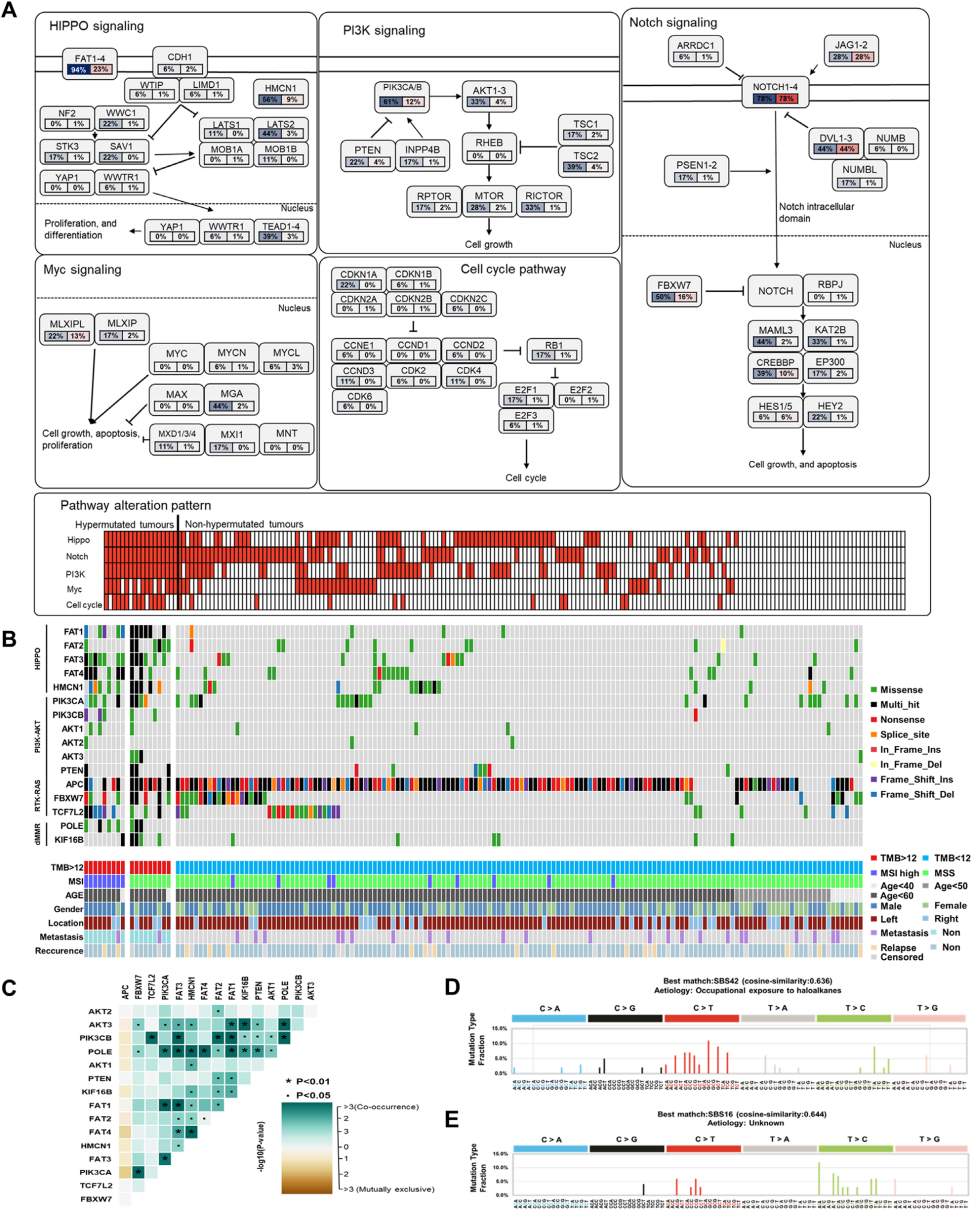
Characteristics of somatic mutations in hypermutated and nonhypermutated CRC. (A) Pathway diagrams showing mutation frequencies in individual genes. Red indicates a higher proportion of mutated patients; solid and dotted lines represent the cell and nuclear membranes, respectively. Arrows indicate activation, bars indicate inhibition, and dotted arrows denote indirect effects. Each cell represents one patient, and red shading indicates at least one alteration in the pathway. (B) Oncoplot of somatic mutations by group. Genes are ordered by pathway (Hippo, PI3K–AKT, RTK–RAS, and dMMR‐related genes). Mutation types are color coded, and corresponding clinical characteristics (TMB, MSI status, age, sex, tumor location, metastasis, relapse) are shown below. (C) Somatic interactions between genes. Cyan indicates co‐occurrence, and brown indicates mutual exclusivity. (D) Mutational signatures identified in the hypermutated group. The *Y*‐axis shows the fraction of mutation types, and the *X*‐axis indicates 96 base substitutions. (E) Mutational signatures identified in the nonhypermutated group. The *Y*‐axis shows the fraction of mutation types, and the *X*‐axis indicates 96 base substitutions.

Hypermutation and MSI are important factors in predicting prognosis and making treatment decisions in CRC. It is known that hypermutated CRCs account for approximately 15–20% of cases, and the causes are often related to MSI, deficient mismatch repair (dMMR), apolipoprotein B mRNA editing enzyme, catalytic polypeptide, *POLE*, and other factors [30]. To evaluate the cause of significant differences in mutation rates, we defined and evaluated hypermutation as TMB > 12 and found that 18 out of 197 cases (9%) were hypermutated, with a median TMB of 34.2. Differences were observed in the HIPPO, PI3K–AKT, NOTCH, MYC, and cell cycle pathways between the two groups (Figure 3A). We also evaluated genes within each pathway that showed significant differences between the two groups. These genes included *FAT1‐4*, *HMCN1*, *PIK3CA*, *PIK3CB*, *AKT1‐3*, *PTEN*, and *APC*, all of which were found to co‐occur, except for *APC* (Figure 3B,C). In the mutational signature analysis of the two groups, single base substitution (SBS) 42 and SBS9 were the most similar signature patterns in the hypermutated group, with cosine similarities of 0.636 and 0.552, respectively, while SBS9 and SBS16 were the most similar signature patterns in the nonhypermutated group, with cosine similarities of 0.516 and 0.644, respectively (Figure 3D,E). The list of mutations associated with MSS hypo mutated CRC appears in Table S4. Previous studies have reported a correlation between the *POLE* P286R mutation and hypermutation (TMB > 100), and our study also showed that all three cases with the *POLE* P286R mutation had TMB > 100 [31]. Interestingly, our study found that the *KIF16B* R145Q mutation significantly co‐occurred with the *POLE* P286R mutation and was found only in the three cases with the *POLE* P286R mutation.

### 2.4 *KRAS*‐Mutant CRCs Exhibit Pronounced Co‐alteration Patterns

**FIGURE 4:**
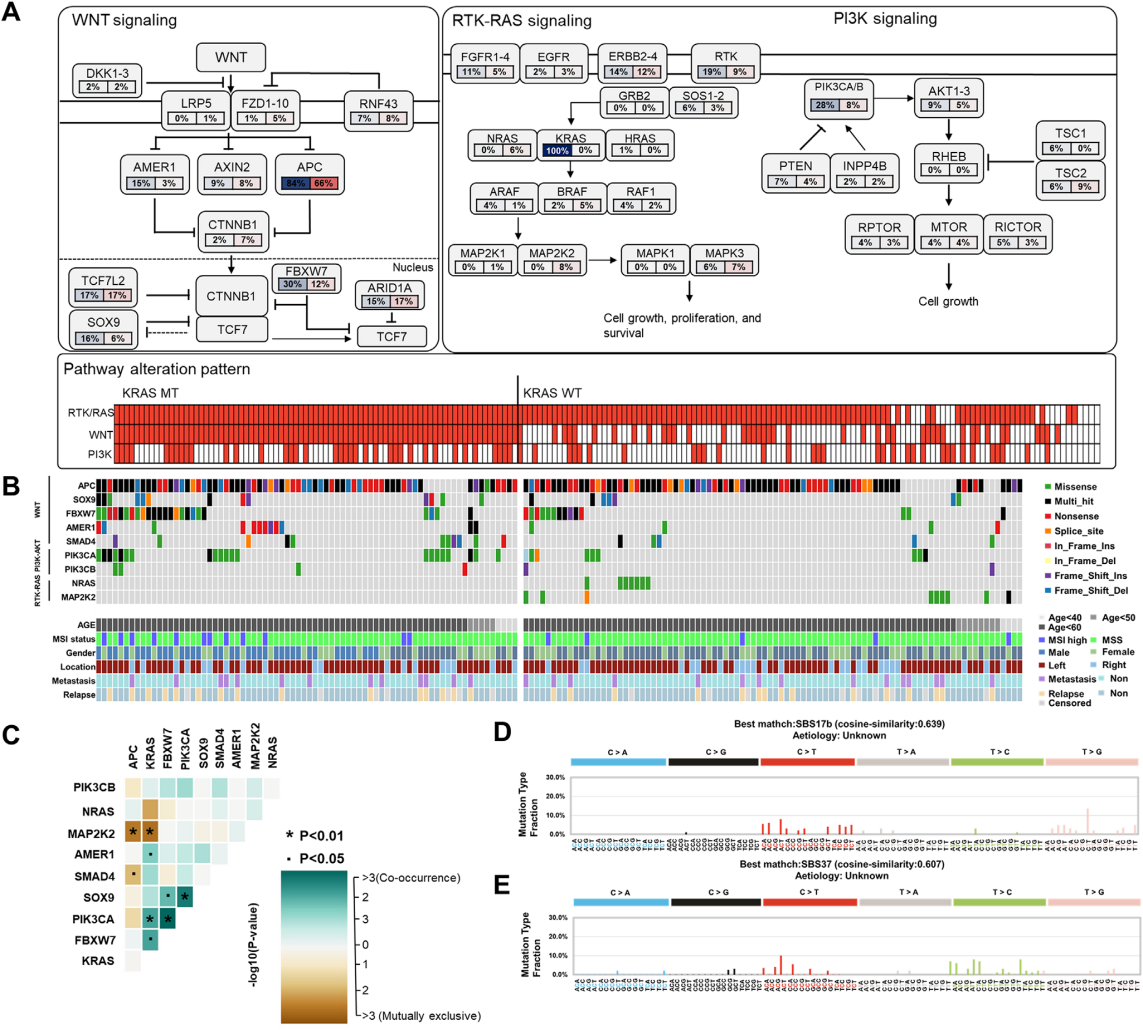
Somatic mutational characteristics of KRAS‐mutated and non‐KRAS CRCs. (A) Pathway diagrams illustrating mutation frequencies in individual genes. Red shading indicates a higher proportion of patients with mutations. Solid and dotted lines represent the cell and nuclear membranes, respectively; arrows indicate activation, bars indicate inhibition, and dotted arrows denote indirect effects. Each cell represents one patient, and red indicates the presence of at least one alteration in the pathway. (B) Oncoplot showing somatic mutation profiles for each group. Genes are ordered by pathway: WNT, PI3K–AKT, and RTK–RAS. Mutation types are color coded, and the lower panel displays clinical characteristics, including age, MSI status, sex, tumor location, metastasis, and relapse status. (C) Somatic interaction matrix of recurrently altered genes. Cyan indicates co‐occurrence, whereas brown indicates mutual exclusivity. (D) Mutational signatures identified in the KRAS‐mutated CRC group. The *Y*‐axis shows the fraction of mutation types, and the *X*‐axis represents 96 base substitution contexts. (E) Mutational signatures identified in the non‐KRAS CRC group. The *Y*‐axis shows the fraction of mutation types, and the *X*‐axis represents 96 base substitution contexts.

*KRAS* mutations are very common in CRC, particularly at codons 12, 13, and 61 of the *KRAS* gene [32]. In our study of 197 Korean CRC patients, 41% had *KRAS* mutations, primarily at codons 12, 13, and 61, with codon 12 being most frequent. Some patients also had mutations at codons 146 and 117, and one patient had multiple mutations. Comparing patients with and without *KRAS* mutations, significant differences were found in oncogenic pathways like WNT, RTK–RAS, and PI3K–AKT, involving key genes *APC, SOX9, FBXW7, AMER1, SMAD4, PIK3CA, PIK3CB, NRAS*, and *MAP2K2* (Figure 4A,B). Additionally, most of the genes showed co‐occurrence, while *KRAS* and *NRAS*, *MAP2K2* were mutually exclusive (Figure 4C). The *KRAS* group and the non‐*KRAS* group were most similar to SBS17b (cosine similarity 0.639) and SBS37 (cosine similarity 0.67), respectively (Figure 4D,E). The *PIK3CA* codon 545 mutation is the most common mutation in *PIK3CA*, and out of the 22 patients with this mutation, 21 also had *KRAS* codon 12 mutations.

### 2.5 Clinical Features Define Distinct Mutational Landscapes in CRC

**FIGURE 5:**
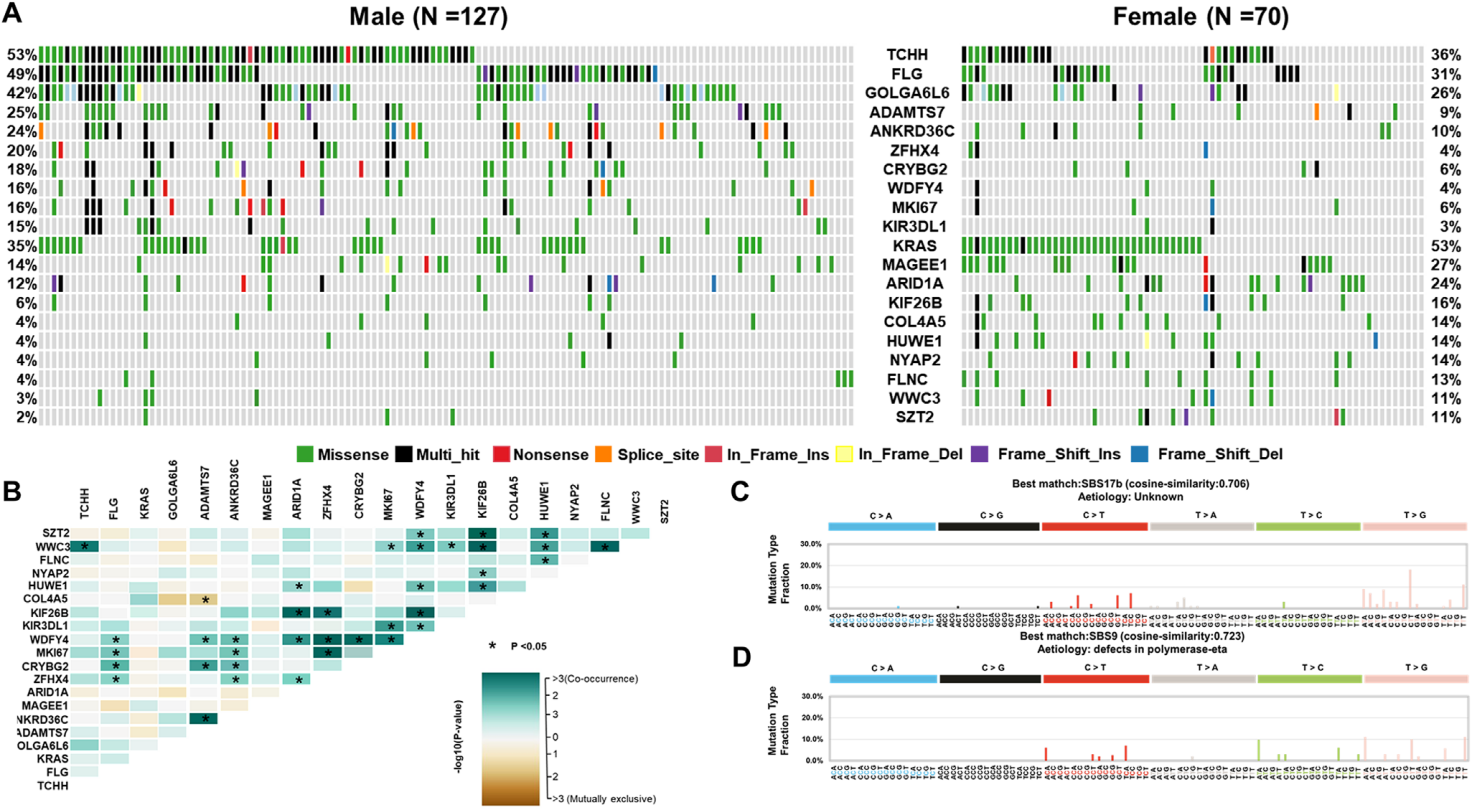
Sex‐based landscape of somatic mutations in CRC patients: comparison of 127 males versus 70 females. (A) The oncoplot depicts the top 10 most frequent variant genes in each group. Each color is indicated according to the mutation type. (B) Somatic interactions between groups. Mutually exclusive or co‐occurring sets of genes were detected using a pairwise Fisher's exact test to detect significant gene pairs. (C) Mutational signatures plots known as SBS in 127 male patients with CRC. (D) Mutational signatures plots known as SBS in 70 female patients with CRC.

**FIGURE 6:**
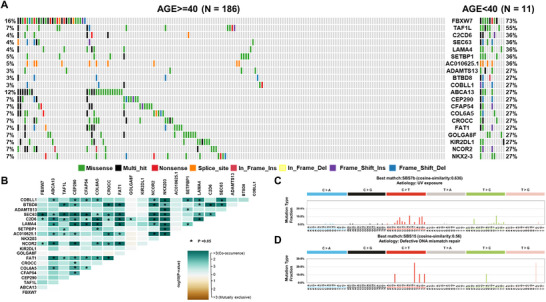
Early‐onset‐based landscape of somatic mutations in CRC patients: comparison of 186 over 40 years old versus 11 under 40 years old. (A) The oncoplot depicts the top 10 most frequent variant genes in each group. Each color is indicated according to the mutation type. (B) Somatic interactions between groups. Mutually exclusive or co‐occurring sets of genes were detected using a pairwise Fisher's exact test to detect significant gene pairs. (C) Mutational signatures plots known as SBS in 186 patients with CRC over 40 years old. (D) Mutational signatures plots known as SBS in 11 patients with CRC under 40 years old.

We explored mutation differences by sex, location of onset, and age in 197 CRC patients. The specific mutations corresponding to each clinical feature are listed in Table S5. Females exhibited higher frequencies of mutations in *KRAS* (53 vs. 35%, *p* value = 0.004**), *MAGEE1* (27 vs. 14%, *p* value = 0.01*), *ARID1A* (24 vs. 12%, *p* value = 0.01*), *HUWE1* (14 vs. 4%, *p* = 0.001***), and *WWC3* (11 vs. 3%, *p* value = 0.02*) compared with males (Figure 5A). Moreover, most of the genes showed co‐occurrence, and in males, SBS17b (cosine similarity 0.706) and SBS9 (cosine similarity 0.723) were the most similar mutational signatures (Figure 5B–D). The top five oncogenic pathways that exhibited significant differences in mutation rates between sexes are shown in Figure S2. Left‐sided CRC had higher prevalence of *TP53* (75 vs. 49%, *p* value = 0.008**) and *APC* (79 vs. 57%, *p* value = 0.004**) mutations compared with right‐sided CRC. *TP53* mutations were mutually exclusive with other genes in left‐sided CRC, while genes in right‐sided CRC tended to co‐occur. Left‐sided CRC resembled SBS5 (cosine similarity 0.615), whereas right‐sided CRC resembled SBS15 (cosine similarity 0.612). Right‐sided CRC also showed higher *POLE* gene mutation frequency and average TMB (Figure S3). Significant differences in mutation rates by cancer site are depicted in Figure S4. Age‐related differences were most pronounced in patients under 40 years old, with diminishing prominence as age increased. In patients under 40 years old, *FBXW7* (73%), *TAF1L* (55%), and *C2CD6* (36%) exhibited higher mutation rates compared with those over 40 years old (Figure 6A), and most of these genes tended to co‐occur (Figure 6B). SBS7b (cosine similarity 0.636) and SBS15 (cosine similarity 0.56) were the most similar mutational signatures in patients under 40 and over 40 years old, respectively (Figures 6C,D and S5). In patients under 50 years old, *FBXW7* (32%), *NBPF1* (35%), and *BSN* (38%) exhibited high mutation rates. These genes tended to co‐occur, though less prominently compared with patients under 40 years old. The most similar mutational signatures were SBS17b (cosine similarity 0.748) for patients under 50 years of age and SBS1 (cosine similarity 0.53) for those over 50 years of age (Figures S6 and S7). We analyzed mutation patterns in 197 patients based on recurrence and metastasis. Recurrence (18 patients) showed mutations in *APC* (72%), *TP53* (67%), and *KRAS* (44%). Nonrecurrence (145 patients) had *APC* (77%), *TP53* (68%), and *KRAS* (41%) mutations. Metastasis (34 patients) displayed high mutation rates in *TP53* (77%), *APC* (63%), and *KRAS* (54%), with specific mutations in *MUC17*, *SALL2*, and *CELA3A*, particularly in liver metastasis cases (Figures S8 and S9).

## 3 Discussion

CRC exhibits substantial genomic heterogeneity, comprising a few recurrent driver mutations and a wide spectrum of low‐frequency alterations [33]. Landmark genomic studies such as those from TCGA and the International Cancer Genome Consortium have delineated key hallmarks of CRC, including recurrent mutations in *APC*, *TP53*, and *KRAS*, while underscoring significant inter‐patient variability [31]. However, these datasets are predominantly based on individuals of European ancestry, and genomic data from East Asian populations remain underrepresented. Prior studies have highlighted clinical and molecular discrepancies between Western and East Asian CRC patients—including differences in mutation frequencies, MSI‐H prevalence, and treatment responses—emphasizing the necessity for population‐specific genomic analyses [33, 34]. Moreover, genetic background, lifestyle factors, and epigenetic modifications vary across populations and interact in complex ways to influence cancer development. These population‐specific differences underscore the need for stratified genomic analyses, particularly in underrepresented groups such as East Asians, to elucidate tumorigenic mechanisms and inform the development of precision oncology strategies [35, 36, 37]. In this context, we performed WGS of 197 paired Korean CRC samples, identifying mutation frequencies of *APC* (89%), *TP53* (68%), and *KRAS* (41%), consistent with global CRC profiles. Notably, our WGS approach revealed a set of previously unreported and potentially recurrently mutated genes, including *MAP1A*, *GOLGA6L6*, *CBWD5*, *LRRIO3*, *TRIM64B*, *SPINK5*, and *ZNRF2*, which were absent in TCGA‐COAD/READ and prior Korean WES studies. These findings highlight the unique value of comprehensive WGS in uncovering rare variants and suggest novel avenues for understanding the broader genomic landscape of CRC within East Asian populations.

Recent studies have highlighted that hypermutation in CRC can arise through distinct mechanisms, most notably MSI and somatic mutations in the exonuclease domain of *POLE* [38, 39]. In our cohort, we identified 18 hypermutated cases based on a TMB of more than 12 mutations per megabase. Notably, three of these hypermutated cases were MSS tumors harboring the *POLE* P286R mutation, with each exhibiting an exceptionally high TMB exceeding 100 mutations per megabase. These findings are consistent with prior reports that *POLE*‐mutated MSS CRCs can display an ultramutated phenotype despite lacking dMMR [40, 41]. *POLE* encodes the catalytic subunit of DNA polymerase epsilon, which is crucial for high‐fidelity DNA replication. Mutations in its exonuclease domain impair proofreading activity and result in the accumulation of mutations. Although *POLE* mutations are relatively rare in CRC, occurring in about 1–2% of cases, their clinical significance is increasingly recognized [42]. Studies have demonstrated that *POLE*‐mutated MSS tumors exhibit distinct clinicopathologic features, such as strong CD8+ T cell infiltration and PD‐L1 expression, characteristics typically associated with MSI‐H tumors [43]. These observations suggest that *POLE*‐mutated MSS tumors may represent an immunologically active subset of CRC, potentially responsive to immune checkpoint inhibitors despite their MSS status. Our findings not only reaffirm the distinct nature of *POLE*‐mutated MSS CRCs within the hypermutated spectrum but also emphasize the importance of further stratifying hypermutated tumors beyond MSI status. Interestingly, we also observed the co‐occurrence of *POLE* P286R mutation and *KIF16B* R145Q mutation in three hypermutated MSS cases, a combination that has not been reported in prior studies. As the field of personalized immunotherapy progresses, comprehensive genomic profiling, including screening for *POLE* mutations and their co‐occurring mutations like *KIF16B*, should be integrated into CRC management, particularly for hypermutated MSS tumors with no other actionable alterations.

*KRAS* mutations, particularly at codon 12, are observed in approximately 40–50% of CRC patients and are widely recognized as a major mechanism of resistance to antiepidermal growth factor receptor therapies such as cetuximab and panitumumab [44]. However, recent studies have highlighted the substantial heterogeneity within *KRAS*‐mutant tumors, suggesting that the presence or absence of co‐occurring mutations can significantly influence tumor behavior, prognosis, and therapeutic response [45]. In our Korean CRC cohort, we identified a frequent co‐occurrence of *KRAS* mutations with alterations in *PIK3CA*, *FBXW7*, and *AMER1*, particularly among tumors harboring *KRAS* codon 12 mutations. Notably, *PIK3CA* mutations, especially at exon 9 (codon 545), were significantly enriched, consistent with previous reports that exon 9 mutations are more prevalent in East Asian populations, including Koreans, compared with exon 20 mutations, which are more frequent in Western cohorts [46]. While exon 20 mutations in *PIK3CA* have been linked to poor prognosis in chemotherapy‐refractory metastatic CRC, exon 9 mutations may have distinct functional and clinical implications, warranting further investigation [47]. *FBXW7*, a tumor suppressor gene involved in the proteasomal degradation of oncoproteins such as c‐Myc, was another gene frequently comutated with *KRAS*. Prior studies suggest that 80–86% of CRC cases with *FBXW7* mutations also harbor *KRAS* mutations, and these comutated tumors tend to exhibit more aggressive phenotypes and poorer clinical outcomes. However, it is important to note that these findings are based on studies with relatively small sample sizes, which may limit the generalizability of this association. Further research with larger patient cohorts is needed to confirm these results and better understand the clinical significance of *KRAS*–*FBXW7* comutation in CRC [48]. Similarly, *AMER1*, a negative regulator of the WNT pathway, was comutated in our *KRAS*‐mutant group. Emerging evidence indicates that *KRAS*–*AMER1* comutation is associated with reduced progression‐free survival in metastatic CRC [49]. Interestingly, we also observed *MAP2K2*, a key component of the RAS/RAF/MEK/ERK signaling cascade, showing mutual exclusivity with *KRAS* mutations in our dataset. This finding is consistent with the concept of functional redundancy within the MAPK pathway, where *KRAS* activation might bypass the need for additional mutations downstream, potentially minimizing the selective advantage of *MAP2K2* mutations.

The significance of gender differences in the incidence, prognosis, and treatment response of CRC has been recognized, but our understanding of the underlying causes for these differences is still limited. While several studies have identified clinical and molecular differences based on sex, sex is generally not considered when determining CRC treatment approaches [50]. The heterogeneity observed in previous research findings and a lack of understanding regarding the molecular differences related to sex contribute to the hesitation in considering sex in CRC treatment decisions. Therefore, it is crucial to enhance our understanding of the molecular differences between male and female cancers to guide personalized patient treatment. In our study, we observed a relatively similar trend in the landscape of somatic mutations in autosomal genes when comparing male and female CRC patients. An intriguing finding was that female CRC patients exhibited significantly more mutations in genes associated with the X chromosome. The human genome contains approximately 6% tumor suppressor genes, with around 2% located on the X chromosome, including well‐known mutations such as *BRCA1* and *BRCA2* [51]. This has led to ongoing efforts to enhance our understanding of mutations in X‐linked genes. Most X‐linked genes are present in a hemizygous state on the X chromosome, making them susceptible to single‐hit genetic inactivation [52]. Biallelic irregularities in genes involved in X chromosome inactivation, particularly escape genes, can increase the risk of cancers in females. Identifying biallelic genes that contribute to X chromosome inactivation is crucial for understanding how mutations in X‐linked genes contribute to cancer development [53]. In our study, we found that female CRC patients had a significantly higher number of mutations in X‐linked genes, including *MAGEE1*, *WWC3*, and *HUWE1*, compared with males, in addition to mutations in *KRAS* and *ARID1A*. Furthermore, *MAGEE1* is frequently mutated and considered a candidate cancer gene in breast cancer, *WWC3* has a high potential for complete or partial escape in ovarian cancer, and *HUWE1* is known as a tumor suppressor gene [54]. Therefore, gaining a better understanding of gene mutations in X‐linked genes can provide valuable insights into the underlying causes of gender‐specific differences in CRC.

EOCRC, typically defined as CRC diagnosed before the age of 50 years, is characterized by more aggressive clinical behavior compared with its later‐onset counterparts. Patients with EOCRC often present with poor differentiation, lymphovascular invasion, and are diagnosed at more advanced stages, leading to a poorer prognosis [55]. Despite these aggressive clinical features, the molecular mechanisms underlying EOCRC remain incompletely understood, with MSI‐H and dMMR being common but not universal in these patients [56]. In our cohort, we found that *FBXW7* mutations were significantly more prevalent in younger patients, particularly those under 40 years old. This finding aligns with previous studies suggesting that *FBXW7* mutations may be a common feature of EOCRC, contributing to its aggressive nature [57]. *FBXW7* functions as a tumor suppressor by targeting oncogenic substrates, such as c‐Myc and Notch, for degradation. The inactivation of *FBXW7* may enhance the WNT/β‐catenin pathway, which is known to play a crucial role in tumor progression by promoting cell proliferation and survival [58]. Furthermore, we observed that *SEC63*, a gene involved in endoplasmic reticulum homeostasis and potentially in regulating WNT/β‐catenin signaling, was frequently comutated with *FBXW7*. *SEC63* mutations have been previously implicated in EOCRC and may influence β‐catenin nuclear translocation under endoplasmic reticulum stress conditions [59]. The concurrent disruption of both *FBXW7* and *SEC63* in EOCRC could synergistically drive WNT pathway hyperactivation, contributing to the distinct molecular phenotype of these tumors. Emerging evidence from studies focusing on WNT/β‐catenin pathway activation in EOCRC suggests that this pathway plays a pivotal role in the disease's pathogenesis. For instance, recent studies have highlighted that WNT signaling is significantly more active in EOCRC compared with later‐onset CRC, further supporting the idea that WNT pathway hyperactivation contributes to the aggressive nature of EOCRC [60, 61]. These findings align with research suggesting that WNT/β‐catenin signaling is a major driver of tumor progression in younger CRC patients, emphasizing the unique molecular characteristics of EOCRC. Targeting WNT/β‐catenin signaling could provide a promising therapeutic approach for EOCRC, particularly for patients with *FBXW7* and *SEC63* mutations, which are associated with enhanced WNT pathway activation and contribute to the aggressive behavior of this disease.

Efforts are ongoing to identify the genetic causes underlying recurrence [62] and metastasis [63] in CRC. In this study, we identified frequent mutations in recurrent CRC patients. *AMER1*, a tumor suppressor gene involved in WNT pathway regulation, is frequently mutated in CRC [2]. Unlike in mesenchymal tumors, *AMER1* mutations in epithelial lineages, including CRC, do not activate the WNT pathway. Mutations in the *AMER1*, *EPHB6*, *FAM153A*, and *PCDH9* genes were observed more than four times in the recurrence group compared with the nonrecurrence group. In contrast, the *TCAF2C*, *PCLO*, and *CES1* genes were only present in the nonrecurrence group. Among these genes, mutations in the *PCLO* gene have been reported in EOCRC, which occurs before the age of 50 years [64]. Therefore, further analysis is required to understand the relationship between these nonrecurrence‐related genes and CRC. Interestingly, contrary to previous studies [65], *KRAS* mutations were more frequently observed in the metastasis group of Korean CRC. Recent evidence suggests that *KRAS* mutations are strongly linked to the development of metastasis in specific sites, particularly the lung (62%; 31 out of 50) and brain (56.5%; 26 out of 46), among patients with CRC [66]. On the other hand, in Korean metastatic CRC, there was a similar high association with lung metastasis (31.6%; six out of 19), and no brain metastasis was observed, but there was a high association with liver metastasis (36.8%; seven out of 19). These results suggest a unique functional role of *KRAS* mutations in Korean metastatic CRC.

This study has several limitations. First, although our cohort represents one of the most comprehensive WGS analyses of Korean CRC patients, the overall sample size (*n* = 197) is relatively modest compared with large international consortia. This may limit the detection of rare mutational events or subtle subtype‐specific differences, reducing the generalizability of our findings to the broader CRC population. Second, clinical data such as treatment history, recurrence status, and long‐term survival outcomes were incomplete in some cases, constraining our ability to robustly correlate genomic alterations with clinical endpoints. Third, although several potentially novel recurrent mutations were identified, their functional roles in CRC progression remain to be validated through experimental or integrative multiomics approaches. Last, while comparisons with publicly available datasets were performed, direct validation in independent Korean cohorts is necessary to confirm the population‐specific relevance of our findings.

## 4 Conclusion

In this study, we performed WGS of 197 paired CRC and matched normal tissue samples from Korean patients, revealing a unique mutational landscape. While common driver mutations in *APC*, *TP53*, and *KRAS* aligned with Western cohorts, we identified novel recurrent mutations not previously reported in TCGA or East Asian datasets. Our findings highlight the molecular heterogeneity of *KRAS*‐mutant tumors, frequent co‐occurrence with *PIK3CA* and *FBXW7* mutations, and age‐specific enrichment of *FBXW7* in EOCRC. These results emphasize the importance of population‐specific genomic profiling for precision oncology. Future validation in independent cohorts and integration with clinical data are needed to translate these findings into therapeutic strategies.

## 5 Materials and Methods

### 5.1 Patients and Tissues

The clinical characteristics of our included cohort of 197 CRC patients are summarized in Table 2. The cohort comprised samples registered in the Clinical & Omics Data Archive (CODA) of the National Biobank of Korea. Fresh‐frozen tissues were obtained from surgically resected primary tumors of 197 patients with CRC who underwent curative surgery between September 1999 and August 2004. The exclusion criteria included inherited syndromes related to cancer and synchronous malignancies, as well as patients who were lost to follow‐up. These tissue specimens, along with patient clinical data, and records of clinicopathological features of the CRC were collected. The baseline clinicopathological characteristics and clinical outcome data were retrospectively collected from the CRC Database of the Department of Colorectal Cancer Surgery and the Pathological Diagnosis Database of the Department of Pathology. All available medical records related to CRC were reviewed to extract clinical information, including the American Joint Committee on Cancer primary tumor, lymph node, distant metastasis classification, the numbers of positive and negative lymph nodes harvested, tumor location, and cause of death in deceased individuals.

**TABLE 2:** Characteristics of patients with Korean CRC.

Variable	*N*	Mean (SD^a^)
Age (years)	197	60.15 (12.03)
	*N*	Percentage (%)
Sex	197	
Female	70	35.53
Male	127	64.47
Location	201	
Ascending	31	15.42
Transverse	11	5.47
Descending	14	6.97
Sigmoid	82	40.80
Rectal	41	20.40
Rectosigmoid	15	7.46
Cecum	6	2.99
Other	1	0.49
Pathology	197	
Adenocarcinoma	187	94.92
mucinous carcinoma	5	2.54
Other	5	2.54
MSI status	197	
MSS	172	87.31
MSI‐low	13	6.60
MSI‐high	11	5.58
Unknown	1	0.51
Primary disease stage	197	
II‐A	60	30.45
II‐B	10	5.08
II‐C	2	1.02
III‐A	4	2.03
III‐B	62	31.47
III‐C	15	7.61
IV‐A	16	8.12
IV‐B	8	4.06
IV‐C	10	5.08
Unknown	10	5.08
Metastasis	48	
Liver	19	39.59
Lung	10	20.83
Lymph node	7	14.58
Bone	1	2.08
Peritoneal	9	18.75
Brain	0	0
Other	2	4.17
Adjuvant chemotherapy	197	
No	52	26.40
Yes	126	63.96
Unknown	19	9.64
Adjuvant regimen	122	
FL	5	4.10
xeloda	8	6.56
FOLFOX	61	50.00
XELOX	48	39.34
Preoperative radio	178	
No	175	98.31
Yes	3	1.69
Postoperative radio	178	
No	173	97.19
Yes	5	2.81
Relapse	197	
No	145	73.60
Yes	18	9.14
Unknown	34	17.26
Death	196	
No	192	97.96
Yes	4	2.04

^a^ SD: standard deviation.

### 5.2 DNA Extraction and WGS

The tumor and matched normal fresh‐frozen tissues obtained from CRC patients were dissected for 20–40 mg and genomic DNAs (gDNAs) were extracted using PureLink Genomic DNA Mini Kit (K182000; Invitrogen, Waltham, MA, USA) by following the manufacturers protocol. Total DNA concentration was calculated using QuantiFluor dsDNA System (E2671; Promega, Madison, WI, USA). To assess the integrity of the gDNAs, samples were analyzed using Genomic DNA ScreenTape Analysis (5067‐5366; Agilent, Santa Clara, CA, USA) with the 4200 TapeStation System (G2991BA; Agilent). The sequencing libraries were prepared according to the manufacturer's instructions of TruSeq Nano DNA High Throughput Library Prep Kit (20015965; Illumina, Inc., San Diego, CA, USA). The paired end (2 × 150 bp) sequencing data were generated using the NovaSeq (20012850; Illumina, Inc.) up to target depths of 30× and 60× for normal and tumor samples, respectively.

### 5.3 Somatic Variant Calling

We analyzed the somatic variants using the DNA pipeline of Illumina DRAGEN Bio‐IT Platform (https://www.illumina.com/products/by‐type/informatics‐products/dragen‐bio‐it‐platform.html). The DRAGEN Bio‐IT Platform integrates all processing from mapping/alignment to the haplotype variant calling into one pipeline. The resulting somatic variant calls were annotated using FUNCOTATOR. To create high‐confidence variant sets, we filtered out variants with the following characteristics: (1) variant allele frequency of 1% or higher in the panel of normals, (2) three or more mismatched bases in the variant reads, (3) common artifact and germline variant sites, (4) frequent presence of error reads in other clones, (5) depth of coverage less than 10 in the tumor sample, and (6) average base quality score below 20 [67, 68, 69].

### 5.4 Analysis of Somatic Variant

The mutation annotation file was loaded into R using the maftools package [70]. The “oncoplot” function provided by maftools was used for group comparison, and the “somaticInteraction” function was used to identify mutually exclusive or co‐occurring gene pairs using pairwise Fisher's exact test. Genes that were differentially mutated between the two groups with a *p* value less than 0.05 were considered for subsequent analysis. Mutational signature analysis was performed using the “BSgenome.Hsapiens.NCBI.GRCh38” package to form the mutation matrix. NMF function was then used to analyze various values, and the fitness was measured based on cophenetic correlation. The value with the lowest correlation was selected and compared with known signatures in the COSMIC database to identify the mutational signature with the highest cosine similarity. For driver gene analysis, we used the oncodrive function based on the oncodriveFML algorithm to identify driver genes. We defined genes with a false discovery rate of *p* < 0.05 as driver genes. We conducted survival analysis to evaluate the effect of driver genes on recurrence, and performed the analysis using the mafSurvive function.

### 5.5 CNV Analysis

CNV analysis was conducted using the GATK4 CNV pipeline. Segmented copy ratio profiles were generated for each sample using ModelSegments and further processed into discrete CNV calls with CallCopyRatioSegments. Gene‐level annotation of CNV regions was performed by intersecting CNV segments with the GENCODE v38 gene annotation (GTF format) using R. Genes overlapping with amplified or deleted segments were identified based on predefined copy ratio thresholds. To assess the functional impact of CNVs, pathway‐level summaries were generated for selected oncogenic signaling pathways. The frequency of CNV events within each pathway was calculated and compared across all samples.

### 5.6 Statistics Analysis

All statistical analyses were performed using the R software v4.1.2 (https://www.R‐project.org/). Chi‐square tests were employed to evaluate differences in mutation frequencies between cohorts across oncogenic pathways, based on categorical proportion data. The Wilcoxon rank‐sum test (for two groups) or Kruskal–Wallis test (for more than two groups) was used to examine group differences for continuous measures. Fisher's exact test was used to assess the enrichment of mutations in a given gene as compared with the background mutation rate. All tests were two‐sided with *p* < 0.05 being considered statistically significant.

## Author Contributions

Conceptualization: S.K. and Y.H.K. Data curation: J.K., D.M.L., Y.J.K., H.S., T.Y.K., K.J.P., S.B.K., C.S.Y., and J.L.L. Formal analysis: J.K., D.M.L., Y.Y., H.L., E.J.K., H.M.K., S.M., D.K., H.J.H., E.K.K., H.S.L., S.E.B., and Y.H.K. Funding acquisition: Y.H.K. and S.K. Investigation: S.U.B., J.W.P., T.K.K., D.L., K.K., C.K.O., D.S.K., S.C., H.R.P., S.K., and Y.H.K. Methodology: J.K., D.M.L., Y.Y., H.L., E.J.K., H.M.K., S.M., D.K., H.J.H., E.K.K., H.S.L., and S.E.B. Project administration: S.K. Software: J.K. and D.M.L. Supervision: S.K. and Y.H.K. Visualization: J.K. and D.M.L. Roles/writing – original draft: J.K., D.M.L, S.U.B., J.W.P., T.K.K., D.L., K.K., C.K.O., D.S.K., S.C., and H.R.P. Writing – review and editing: S.K. and Y.H.K. All authors listed have consented to the submission and publication of this manuscript. They have reviewed the final version of the manuscript and agree to its content.

## Ethics Statement

This study was conducted in accordance with the Declaration of Helsinki (as revised in 2008) and was approved by the Institutional Review Board of Dongsan Medical Center (No. DSMC 2024‐01‐062). Informed consent was obtained from all participants.

## Conflicts of Interest

The authors declare no conflicts of interest.

## Study Approval

This study was provided with bioresources from CODA (CODA_S2200008‐01) in National Biobank of Korea, the Agency for Disease Control and Prevention, Republic of Korea.

## Funding Information

This work was supported by the National Research Foundation of Korea (NRF) grant funded by the Korea government (MSIT) (RS‐2024‐00406152, RS‐2024‐00439078, RS‐2023‐00249115, 2021M3E5D7102565). This work was supported by the National Research Foundation of Korea (NRF) grant funded by the Ministry of Education (RS‐2025‐02305555). This research was supported by a grant of the Korea Health Technology R&D Project through the Korea Health Industry Development Institute (KHIDI), funded by the Ministry of Health & Welfare, Republic of Korea (RS‐2025‐02223691). This research was also supported by the Bio & Medical Technology Development Program of the NRF funded by the Ministry of Health and Welfare, Ministry of Science and ICT, Ministry of Trade Industry and Energy, and Korea Disease Control and Prevention Agency (The National Project of Bio Big Data) (NRF‐2020M3E5D7085175). This work was supported by KREONET.

## Supporting information



Figure S1. Comparative analysis of somatic variants patterns and oncogenic pathway alterations between Korean CRC and TCGA‐CRC cohorts.
Figure S2. Sex‐specific mutation patterns in top five oncogenic pathways.
Figure S3. Landscape of somatic mutations in CRC patients (149 left CRC vs 47 right CRC).
Figure S4. Left and right CRC‐specific mutation patterns in top five oncogenic pathways.
Figure S5. Mutational patterns in the top 5 oncogenic pathways according to age 40 criteria.
Figure S6. Landscape of somatic mutations in CRC patients (160 over 50 years old vs 37 under 50 years old).
Figure S7. Landscape of somatic mutations in CRC patients (160 over 50 years old vs 37 under 50 years old).
Figure S8. Landscape of somatic mutations in CRC patients based on recurrence and metastasis. (18 recurrence vs 145 nonrecurrence and 34 metastatic CRC).
Figure S9. Mutational patterns in the top 5 oncogenic pathways according to relapse status.
Supplementary Table 1. List of driver genes identified through positional clustering in 197 CRC patients.
Supplementary Table 2. List of mutational hotspots in Korean CRC.
Supplementary Table 3. List of driver single genes and gene combinations associated with prognosis in Korean CRC patients.
Supplementary Table 4. List of mutations that occurred in hypo mutated MSS.

Supplementary Table 5. List of specific mutations according to clinical variables.

## Data Availability

The Korean CRC WGS data can be provided by CODA (https://coda.nih.go.kr/) pending scientific review and a completed material transfer agreement. Requests for the WGS data should be submitted to: access ID (CODA_D22002).
