# Supplementary material for: Characterization of Korean Colorectal Cancer Reveals Novel Driver Gene and Clinically Relevant Mutations
Source: MedComm (2020). 2026 Jan 8;7(1):e70584. doi: 10.1002/mco2.70584 (PMC12783921; doi:10.1002/mco2.70584)
Supplement: Supplementary file 1 — Figure S1. Comparative analysis of somatic variants patterns and oncogenic pathway alterations between Korean CRC and TCGA‐CRC cohorts. Figure S2. Sex‐specific mutation patterns in top five oncogenic pathways. Figure S3. Landscape of somatic mutations in CRC patients (149 left CRC vs 47 right CRC). Figure S4. Left and right CRC‐specific mutation patterns in top five oncogenic pathways. Figure S5. Mutational patterns in the top 5 oncogenic pathways according to age 40 criteria. Figure S6. Landscape of somatic mutations in CRC patients (160 over 50 years old vs 37 under 50 years old). Figure S7. Landscape of somatic mutations in CRC patients (160 over 50 years old vs 37 under 50 years old). Figure S8. Landscape of somatic mutations in CRC patients based on recurrence and metastasis. (18 recurrence vs 145 nonrecurrence and 34 metastatic CRC). Figure S9. Mutational patterns in the top 5 oncogenic pathways according to relapse status. Supplementary Table 1. List of driver genes identified through positional clustering in 197 CRC patients. Supplementary Table 2. List of mutational hotspots in Korean CRC. Supplementary Table 3. List of driver single genes and gene combinations associated with prognosis in Korean CRC patients. Supplementary Table 4. List of mutations that occurred in hypo mutated MSS. [file MCO2-7-e70584-s001.pdf]

Characterization of Korean Colorectal Cancer Reveals Novel Driver Gene and Clinically Relevant Mutations

Running Head: Genomic Profiling of Korean Colorectal Cancer

Junho Kang[1#], Dong Min Lim[2#], Young-Joon Kim[3], Hyeran Shim[3], Tae-You Kim[4], Kyu Joo Park[5], Sung-Bum Kang[6], Chang Sik Yu[7], Jong Lyul Lee[7], Yeuni Yu[8], Hansong Lee[8], Eun Jung Kwon[8], Hyo Min Kim[2], Seongik Mun[2], Donghee Kwak[9], Hae Seul Lee[9], Hye Jin Heo[10], Eun Kyoung Kim[10], Seung Eun Baek[10], Jong-Wook Park[11], Sung Uk Bae[12,13], Taeg Kyu Kwon[11,12,14], Dongjun Lee[9], Kihun Kim[15], Chang-Kyu Oh[16], Dai Sik Ko[17], Sunghwan Cho[18], Hae Ryoun Park[19], Shin Kim[1,11,12*], Yun Hak Kim[9,15*]

[1]Department of Research, Keimyung University Dongsan Medical Center, Daegu, 42601, Republic of Korea.

[2]Medical Research Institute, Pusan National University, Yangsan, 50612, Republic of Korea.

[3]Department of Biochemistry, College of Life Science and Biotechnology, Yonsei University, Seoul, 03722, Republic of Korea.

[4]Department of Internal Medicine, Seoul National University College of Medicine, Seoul National University Hospital, Seoul, 03080, Republic of Korea.

[5]Department of Surgery, Seoul National University Hospital, Seoul National University College of Medicine, Seoul, 03080, Republic of Korea.

[6]Department of Surgery, Seoul National University Bundang Hospital, Seoul National University College of Medicine, Seongnam, 13620, Republic of Korea.

[7]Department of Surgery, Division of Colon and Rectal Surgery, University of Ulsan College of Medicine and Asan Medical Center, Seoul, 05505, Republic of Korea.

[8]Medical Research Institute, Pusan National University Pusan, 46241, Republic of Korea.

[9]Department of Convergence Medicine, School of Medicine, Pusan National University, Yangsan, 50612, Republic of Korea.

[10]Department of anatomy, School of Medicine, Pusan National University, Yangsan, 50612, Korea.

[11]Department of Immunology, School of Medicine, Keimyung University, Daegu, 42601, Republic of Korea.

[12]Institute of Medical Science, Keimyung University, Daegu, 42601, Republic of Korea.

[13]Department of Surgery, Keimyung University Dongsan Medical Center, Daegu, 42601, Republic of Korea.

[14]Center for Forensic Pharmaceutical Science, Keimyung University, Daegu, 42601, Republic of Korea.

[15]Department of Biomedical Informatics, School of Medicine, Pusan National University, Yangsan, 50612, Republic of Korea.

[16]Department of Biochemistry, School of Medicine, Pusan National University, Yangsan, Republic of Korea.

[17] Division of Vascular Surgery, Department of General Surgery, Gachon University Gil Medical Center, Incheon, 21565, Republic of Korea.

[18]Department of Surgery, Pusan National University Yangsan Hospital, Yangsan, 50612, Republic of Korea.

[19]Department of Oral Pathology, School of Dentistry, Pusan National University, Yangsan, Republic of Korea.

[†]These authors contributed equally to this work.

Correspondence:

Shin Kim, M.D., Ph.D.

Department of Immunology, School of Medicine, Keimyung University, Daegu, 42601, Republic of Korea

TEL: +82-53-258-7359

Fax: +82-53-258-7355

Email: god98005@dsmc.or.kr

Yun Hak Kim, M.D., Ph.D.

Department of Anatomy, School of Medicine, Pusan National University, Yangsan, 50612, Republic of Korea

TEL: +82-51-510-8091

Fax: +82-51-510-8049

Email: yunhak10510@pusan.ac.kr

Supplementary figures

Figure S1. Comparative analysis of somatic variants patterns and oncogenic pathway alterations between Korean CRC and TCGA-CRC cohorts.

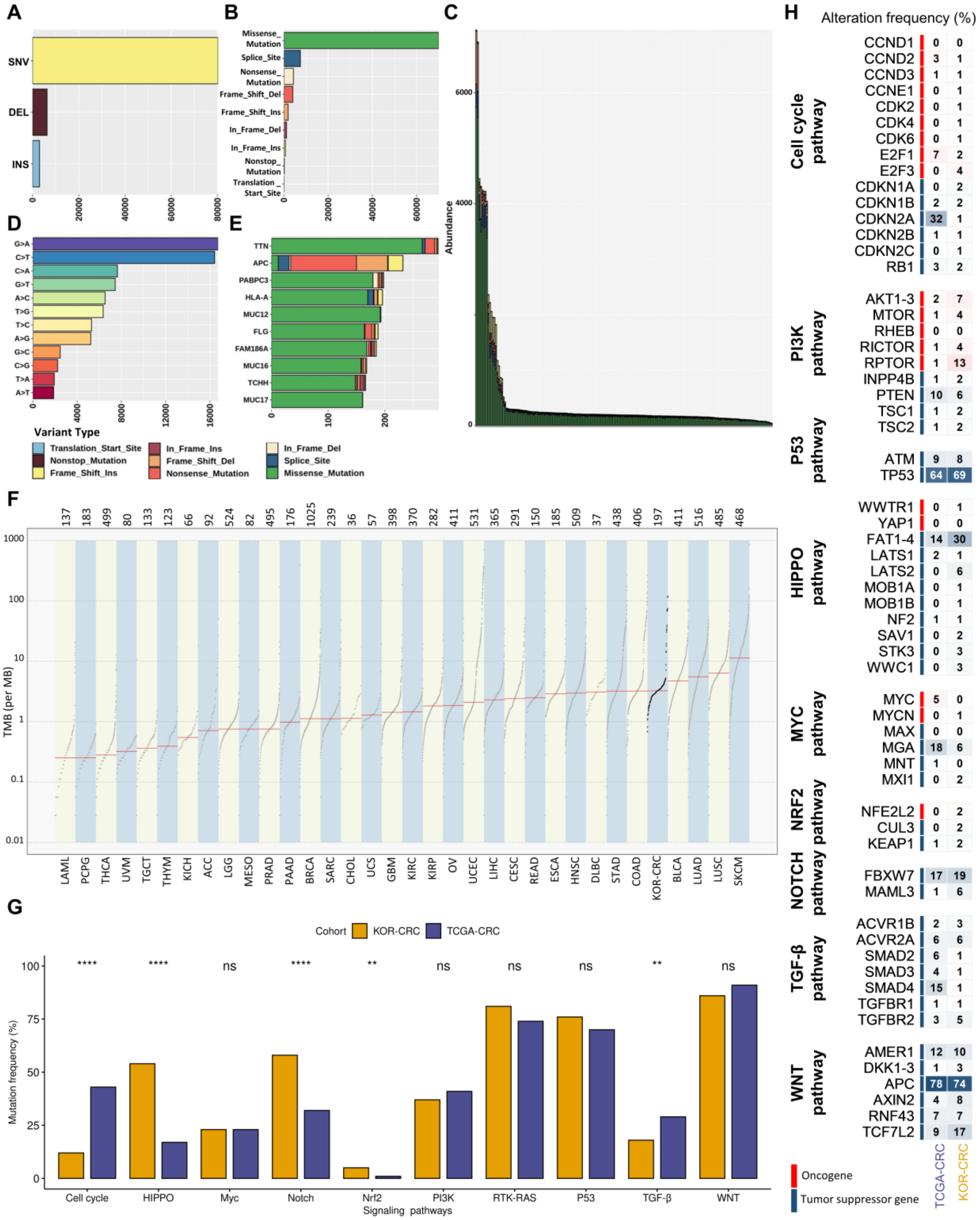

A: SNVs and INDEL in all patients. The x-axis represents the total number of variants, and the

y-axis represents the variant type. The mutation rates according to SNV are shown, with SNV, DEL, and INS indicated. B: Variant classification. The x-axis represents the total number of variants, and the y-axis represents the variant classification. C: Mutation burden of all patients. The y-axis represents the number of mutations for each patient, and the color in each bar plot represents the variant type. Each color is matched with the appropriate legend. D: Changes in bases constituting each variant type. The x-axis represents the number of base changes, and the y-axis represents the number of base changes. E: Genes containing the most mutations in all patients. The x-axis represents the number of mutations contained in each gene, and the y-axis represents the gene with the most mutations in sequence. Each color matches the legend on the bottom side. F: Tumor mutational burden distribution across 33 TCGA cancer types and the Korean CRC cohort. Cancer types are ordered by increasing median TMB. The numbers above each column indicate the number of samples included in each cancer type. G: Comparison of pathway-level mutation frequencies between the Korean CRC and TCGA-CRC cohorts across ten canonical oncogenic signaling pathways. Statistical significance was assessed using chi-square tests. **** $p < 0.0001$; ** $p < 0.01$; ns, not significant. H: Gene-level alteration frequencies within each oncogenic pathway, highlighting differences between TCGA-CRC and Korean CRC cohorts in key oncogenes and tumor suppressor genes. Oncogenes and tumor suppressor genes are indicated by red and blue bars, respectively.

Figure S2. Sex-specific mutation patterns in top five oncogenic pathways.

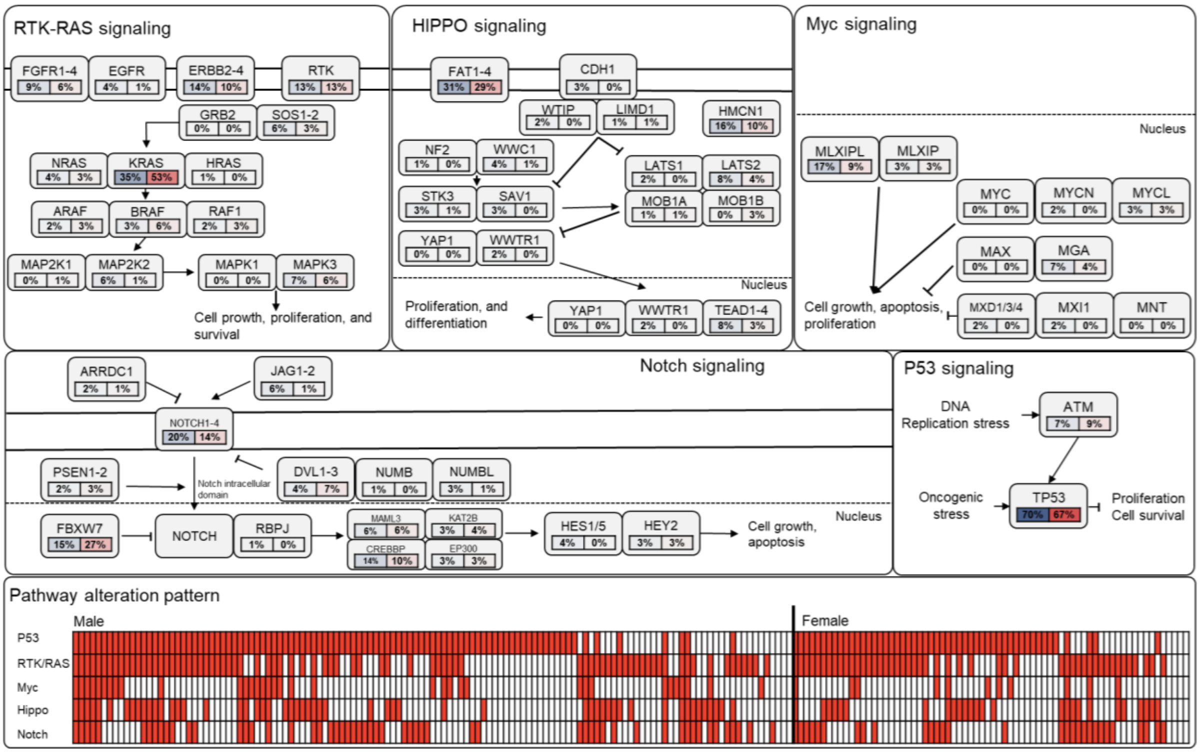

The percentage indicated under each gene represents the proportion of patients with a mutation in that gene among all patients, with red indicating a higher proportion. The left side of each cell represents males and the right side represents females. The solid lines indicate the cell membrane, and the dotted lines indicate the nuclear membrane. Arrow: activation, bar: inhibition, dotted arrow: Indirect effect of stated change. In the pathway alteration pattern, each cell represents one patient, and red color indicates the presence of at least one genetic alteration in the pathway.

Figure S3. Landscape of somatic mutations in CRC patients (149 left CRC vs 47 right CRC).

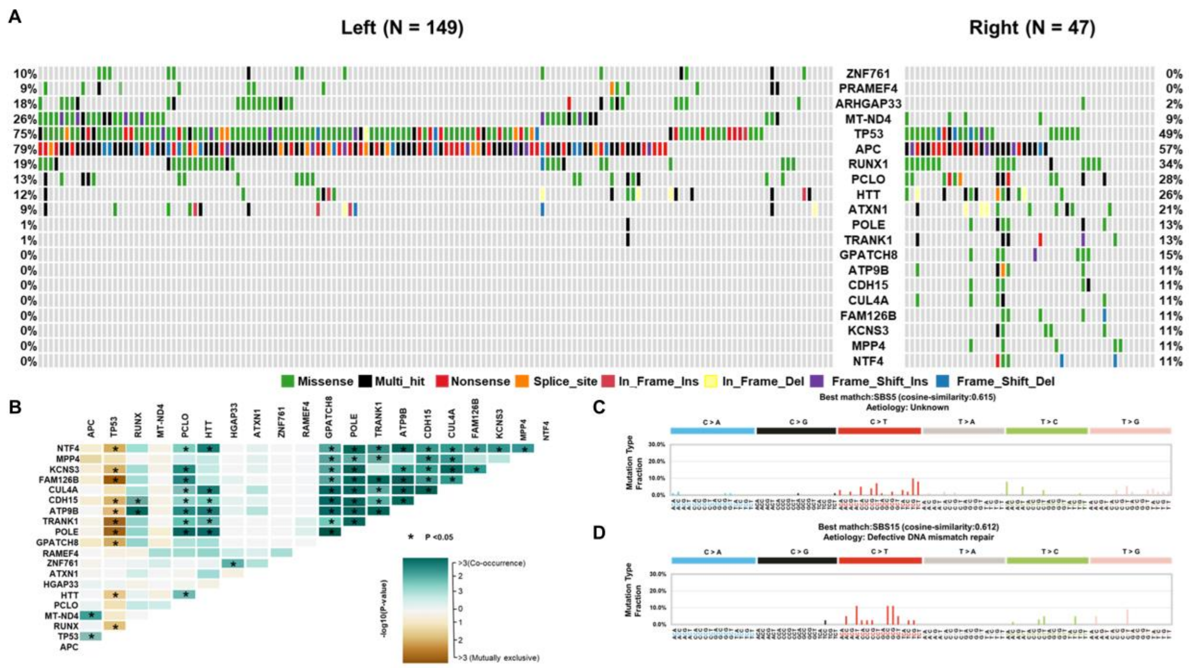

A: The oncoplot depicts the top 10 most frequent variant genes in each group. Each color is indicated according to the mutation type. B: Somatic interactions between groups. Mutually exclusive or co-occurring sets of genes were detected using a pairwise Fisher's exact test to detect significant gene pairs. C: Mutational signatures plots known as SBS in 149 patients with left CRC. D: Mutational signatures plots known as SBS in 47 patients with right CRC.

Figure S4. Left and right CRC-specific mutation patterns in top five oncogenic pathways.

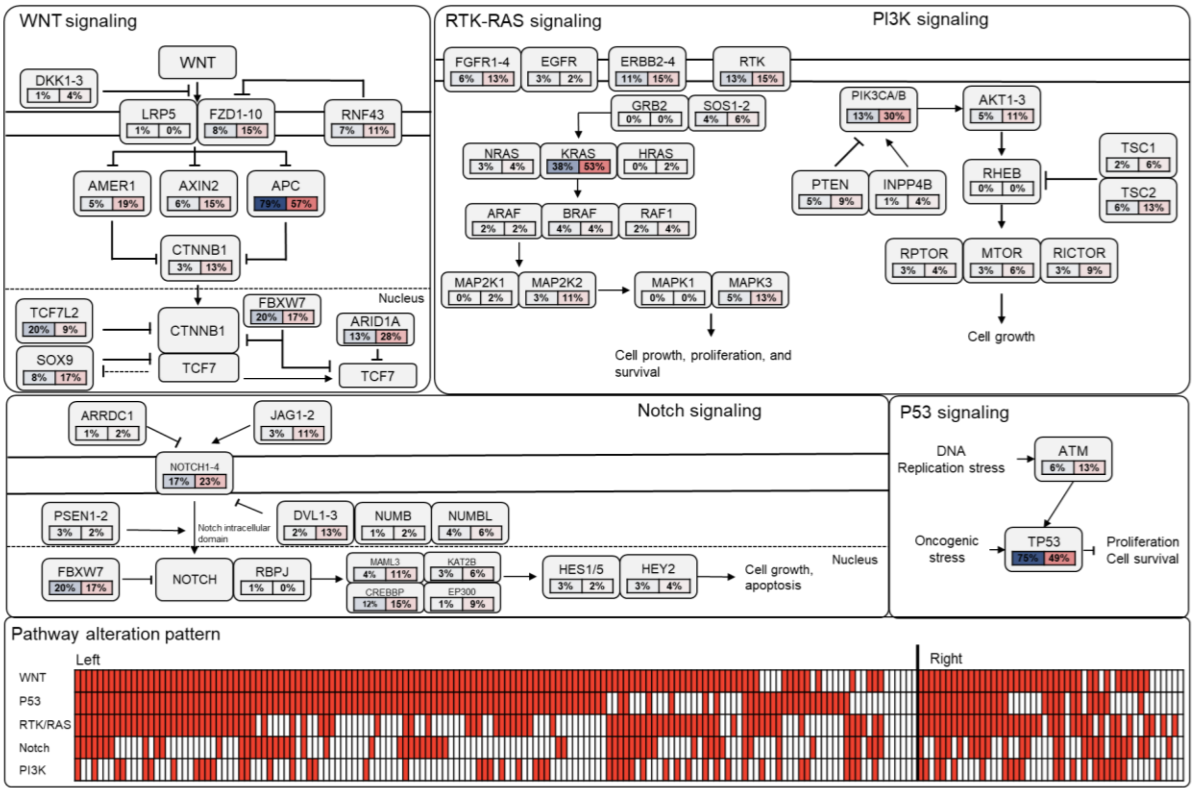

The percentage indicated under each gene represents the proportion of patients with a mutation in that gene among all patients, with red indicating a higher proportion. The left side of each cell represents left-sided CRC and the right side represents right-sided CRC. The solid lines indicate the cell membrane, and the dotted lines indicate the nuclear membrane. Arrow: activation, bar: inhibition, dotted arrow: Indirect effect of stated change. In the pathway alteration pattern, each cell represents one patient, and red color indicates the presence of at least one genetic alteration in the pathway.

Figure S5. Mutational patterns in the top 5 oncogenic pathways according to age 40 criteria.

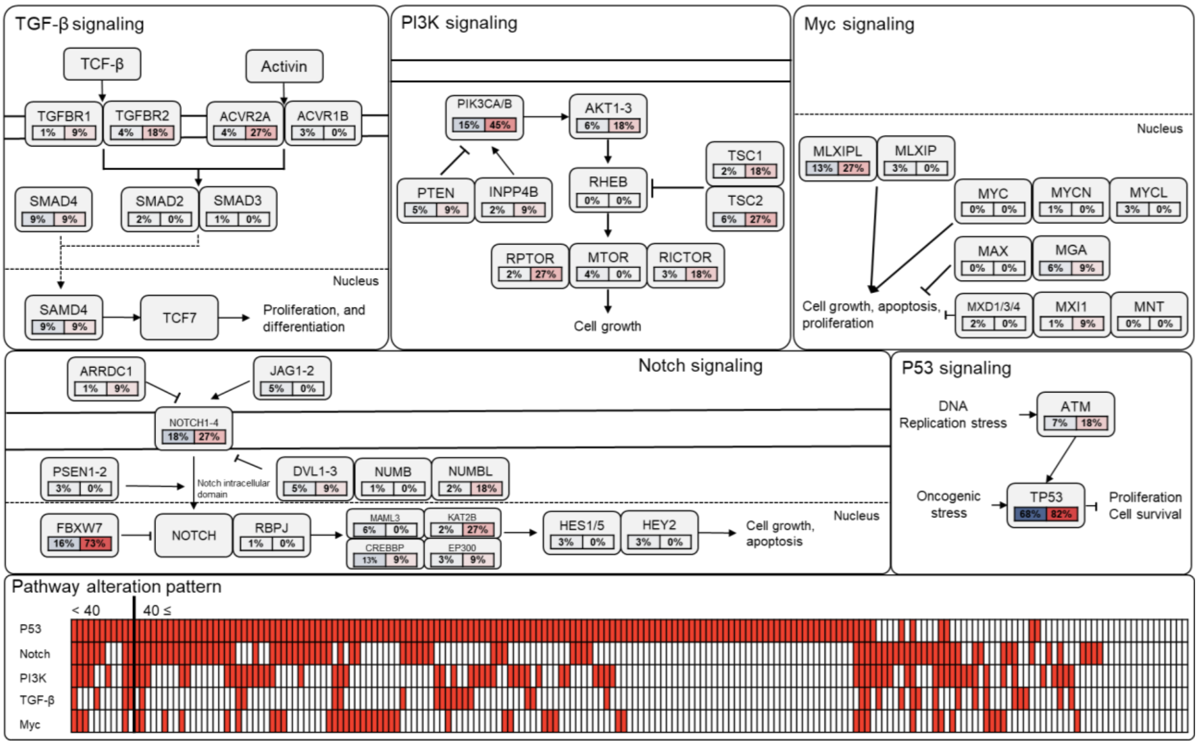

The percentage indicated under each gene represents the proportion of patients with a mutation in that gene among all patients, with red indicating a higher proportion. The left side of each cell represents age <40, and the right side represents 40 or older. The solid lines indicate the cell membrane, and the dotted lines indicate the nuclear membrane. Arrow: activation, bar: inhibition, dotted arrow: Indirect effect of stated change. In the pathway alteration pattern, each cell represents one patient, and red color indicates the presence of at least one genetic alteration in the pathway.

Figure S6. Landscape of somatic mutations in CRC patients (160 over 50 years old vs 37 under 50 years old).

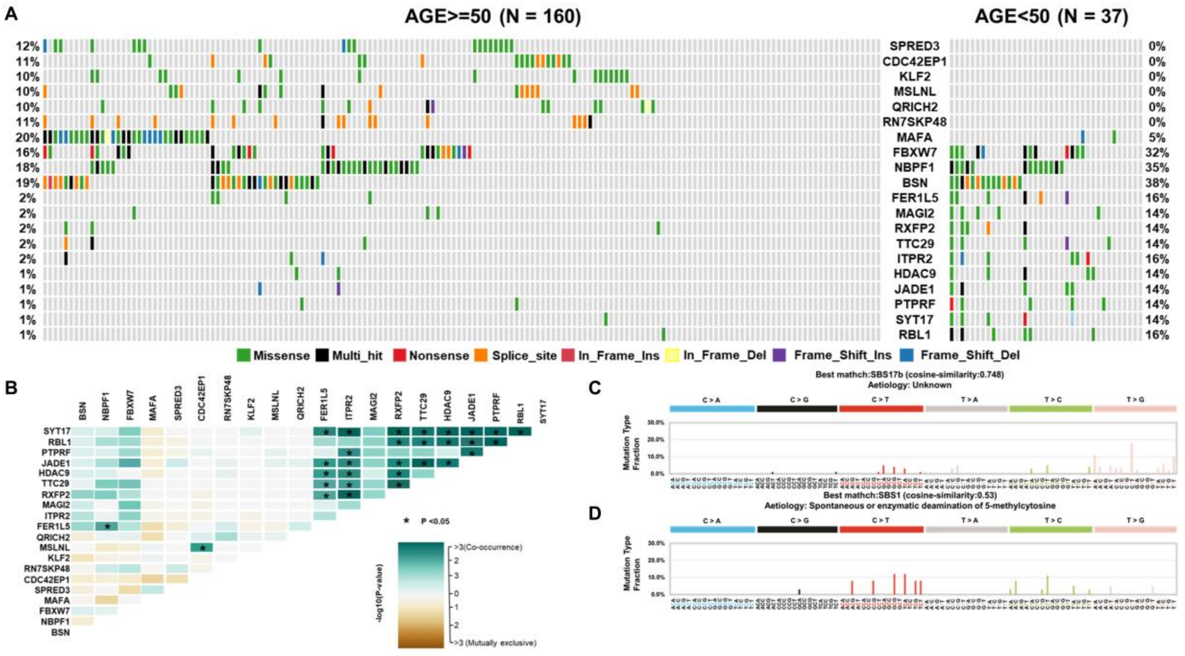

A: The oncoplot depicts the top 10 most frequent variant genes in each group. Each color is indicated according to the mutation type. B: Somatic interactions between groups. Mutually exclusive or co-occurring sets of genes were detected using a pairwise Fisher's exact test to detect significant gene pairs. C: Mutational signatures plots known as SBS in 160 patients with CRC over 50 years old. D: Mutational signatures plots known as SBS in 37 patients with CRC under 50 years old.

Figure S7. Landscape of somatic mutations in CRC patients (160 over 50 years old vs 37 under 50 years old).

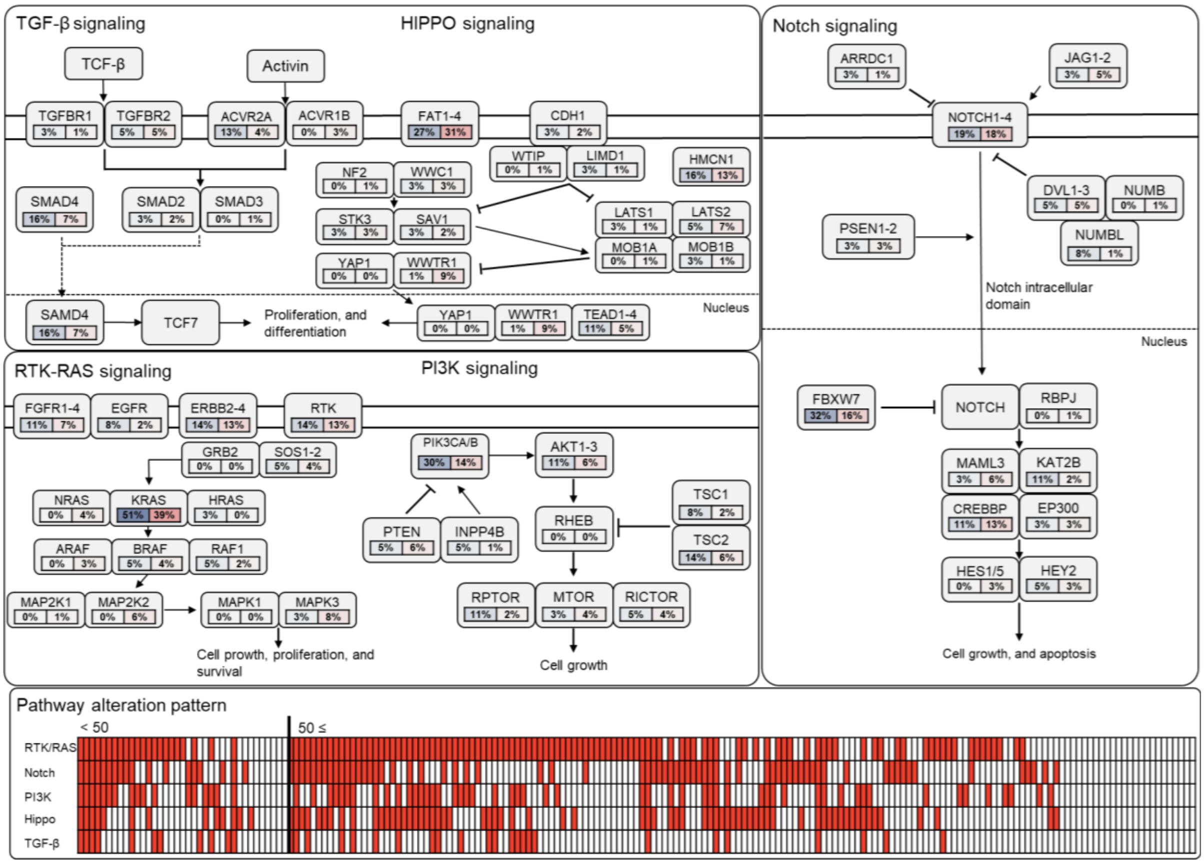

The percentage listed below each gene denotes the proportion of patients with a mutation in that gene among all patients, with red indicating a higher proportion. The left side of each cell represents patients under the age of 50, while the right side represents those aged 50 or older. Solid lines denote the cell membrane, and dotted lines indicate the nuclear membrane. Arrows indicate activation, bars represent inhibition, and dotted arrows signify an indirect effect of the stated change. In the pathway alteration pattern, each cell represents one patient, with red color indicating the presence of at least one genetic alteration in the pathway.

Figure S8. Landscape of somatic mutations in CRC patients based on recurrence and metastasis. (18 recurrence vs 145 non- recurrence and 34 metastatic CRC).

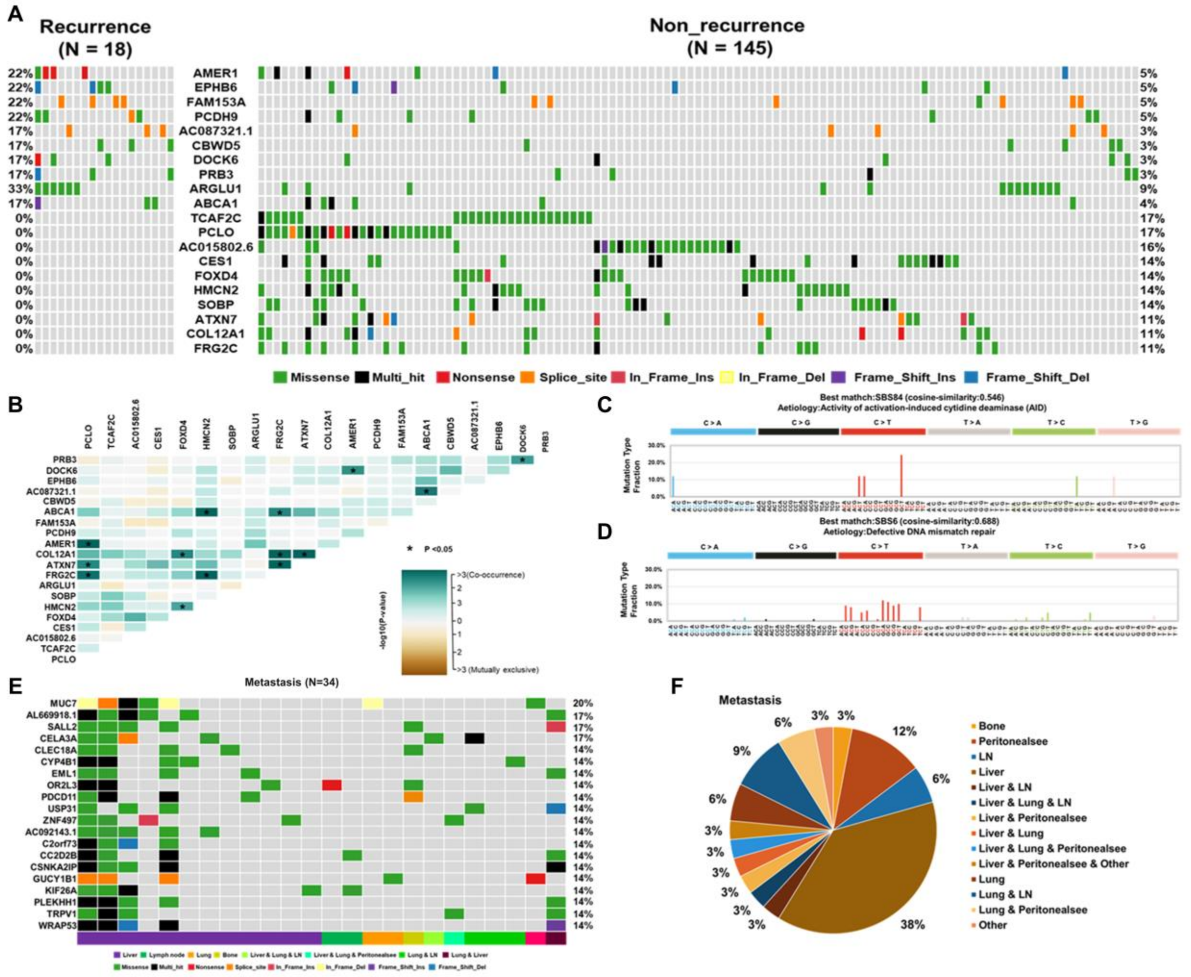

A: The oncoplot depicts the top 10 most frequent variant genes in each group. Each color is indicated according to the mutation type. B: Somatic interactions between groups. Mutually exclusive or co-occurring sets of genes were detected using a pairwise Fisher's exact test to detect significant gene pairs. C: Mutational signatures plots known as SBS in 18 patients with recurrent CRC. D: Mutational signatures plots known as SBS in 145 patients with no recurrence CRC. E: The oncoplot depicts the top 20 most frequent variant genes in patients with metastatic CRC. Each color is indicated according to the primary tumor location in metastatic CRC. F:

Pie chart showing the distribution of primary tumor locations in 34 patients with metastatic CRC.

Figure S9. Mutational patterns in the top 5 oncogenic pathways according to relapse status.

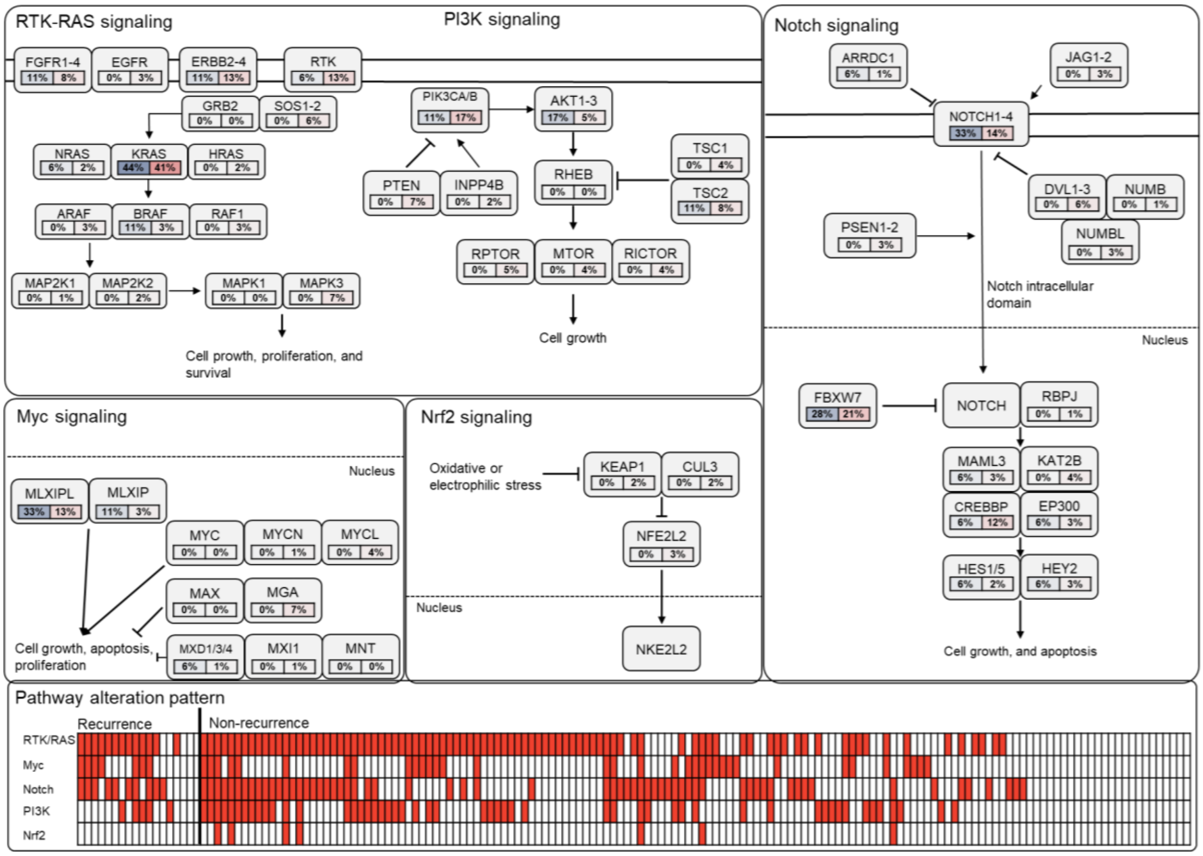

The percentage indicated under each gene represents the proportion of patients with a mutation in that gene among all patients, with red indicating a higher proportion. The left side of each cell represents relapse CRC and the right side represents non-relapse CRC. The solid lines indicate the cell membrane, and the dotted lines indicate the nuclear membrane. Arrow: activation, bar: inhibition, dotted arrow: Indirect effect of stated change. In the pathway alteration pattern, each cell represents one patient, and red color indicates the presence of at least one genetic alteration in the pathway.

Supplementary Table 1. List of driver genes identified through positional clustering in 197 CRC patients.

Gene	Cluster score	FDR
TP53	0.5666714	4.77E-21
KRAS	0.9535657	3.12E-18
HRNR	0.5234591	5.18E-08
ZNF676	0.8518519	5.57E-08
APOL1	0.8472222	1.50E-06
PLIN4	0.7243174	3.69E-06
LILRB2	0.1184211	1.5339802-03
MAP1A	0.96	1.56E-03
TBP	0.8888889	1.57E-03
ESRRA	0.9615385	3.28E-03
CBWD5	0.9166667	7.03E-03
CCDC73	1	1.40E-03
CPEB2	0.8	2.74E-02
CKAP4	0.8	2.74E-02
LMTK3	0.8627961	1.40E-02
ZNF595	0.8	2.74E-02
LILRB3	0.7648759	4.63E-02
DHRS4	0.9444444	4.13E-03
LRRIQ3	0.8333333	2.12E-02
TRIM16	1	1.40E-03
TRIM64B	0.8333333	2.12E-02
UBXN11	1	1.40E-03
KRTAP10-4	0.9047619	8.74E-03
OR10G2	1	1.40E-03
RUNX1	0.8	2.74E-02
TTC33	1	1.40E-03
NBPF9	0.9411765	4.28E-03
TEAD2	1	1.40E-03
ZNF705G	1	1.40E-03
ACVR2A	0.875	1.29E-02
AGAP9	1	1.40E-03
ANXA8L1	1	1.40E-03

ARMCX4	0.9	9.33E-03
ATN1	1	1.40E-03
C1QTNF5	1	1.40E-03
CELA1	0.8	2.74E-02
CENPB	0,8333333	2.12E-02
DDX27	0.8	2.74E-02
EVX2	1	1.40E-03
FTSJ3	0,8000000	2.74E-02
GPRIN1	0.8	2.74E-02
HCAR2	0.8571429	1.65E-02
HLA-DQB1	0.8	2.74E-02
KCNJ18	0.7679517	4.46E-02
KRTAP9-6	0.8828427	1.14E-02
LMO2	0.8333333	2.12E-02
LRRC37A2	0.7894737	3.20E-02
MZF1	0.8946394	1.01E-02
NDUFC2	0.8	2.74E-02
NPHP1	0.8571429	1.65E-02
NPY4R	1	1.40E-03
NRAS	0.9581581	3.28E-03
OR2T2	1	1.40E-03
OR2T29	0,8333333	2.12E-02
PCBP1	1	1.40E-03
PIK3CA	0.7697864	4.39E-02
POU3F3	0.8	2.74E-02
PRRT1	0.8	2.74E-02
RBM33	0.8	2.74E-02
RBMXL 1	0.7941942	2.99E-02
RPL22	1	1.40E-03
SAMD1	1	1.40E-03
SLC23A2	1	1.40E-03
SLC9B1	0.8333333	2.12E-02
SMAD4	0.8333333	2.11E-02
SMC6	1	1.40E-03
SPINK5	0.8	2.74E-02

SPRED3	0.8888889	1.03E-02
SRRT	0.8	2.74E-02
TFAM	1	1.40E-03
THAP5	0,8000000	2.74E-02
TXNDC2	0.8	2.74E-02
VPS37C	0.8888889	1.03E-02
WBP1	1	1.40E-03
XPOT	0.8	2.74E-02
ZMI22	0,8125000	2.74E-02
ZNF780B	0,8571429	2.65E-02
ZNRF2	0.8	2.74E-02

Supplementary Table 2. List of mutational hotspots in Korean CRC.

Gene	dnSNP	N	Chromosome (GRCh38)	Variant type	Alleles	Functional Consequence
KRAS	121913529	49	12:25245350	SNP	C>A, G, T	Missense variant
MAP1A	771126505	24	15:43527658	SNP	T>A, G	Missense variant
TP53	28934578	16	17:7675088	SNP	C>A, G, T	Missense variant
PLIN4	28523592	14	19:4511373	SNP	T>C	Missense variant
-	70943391	14	4:85100823	INS	TAA, TAAA, TAAAA, TAAAAA, ***	-
HDHD5, HDHD5-AS1	1263650512	14	22:17159242	SNV	A>C, G, T	Intron variant
KRAS	112445441	13	12:25245347	SNV	C>A, G, T	Missense variant
ZNF676	201994000	13	19:22181068	SNV	C>A, G, T	Missense variant
APOL1	769299217	13	22:36254978	SNV	G>A, T	Missense variant
APOL1	200136783	12	22:36254947	SNV	T>C	Missense variant
ESRRA	759464632	12	11:64315824	DEL	GGG>-	Inframe indel
FRG1FP	1368579899	12	22:10940599	SNV	G>A, T	Non-coding transcript variant
APOL1	199650512	11	22:36254984	SNV	C>T	Missense variant
FLG	554101492	11	1:152308189	SNV	G>A, C, T	Missense variant
HRNR	768388262	11	1:152213342	DEL	CA>-	Frameshift variant
DHRS4, LOC124903290	772099993	11	14:23966329	SNV	C>A	Non-coding transcript variant, missense variant

CACNA1A	1176428893	11	19:13208898	SNV	T>G	Missense variant
LOC100287792	1227603601	11	20:37683234	DELINS	A, AA, AAA, AAAA, AAAAA, AAAAAA, ***	500B downstream variant
NBPF26	1349857272	11	1:120809853	SNV	A>C, G, T	Missense variant
CBWD5	1411096827	11	9:65690975	SNV	A>C, G, T	Genic upstream transcript variant, genic downstream transcript variant
NBPF9	9442084	10	1:149075662	SNV	G>C, T	Missense variant, non-coding transcript variant
TSPEAR, KRTAP10-4	372822453	10	21:44574089	SNV	G>A	Missense variant, Intron variant
-	776164566	10	12:48529907	DELINS	AAAAAA>-, AAA, AAAA, AAAAA, AAAAAAA, AAAAAAAA ***	-
NBPF26	1159820458	10	1:120809865	SNV	T>A, C, G	Missense variant
MZF1, MZF1-AS1	1475416986	10	19:58562227	SNV	G>C	Missense variant, Intron variant
PABPC3, LOC124900613	150143049	9	13:25097674	DELINS	TGCTGCTGC>-, TGCTGC, TGCTGCTGCTGC, TGCTGCTGCTGCTGC	Inframe insertion
ZNF676	769830637	9	19:22181067	SNV	A>C	Missense variant, genic downstream transcript variant
PRAMEF18	878860150	9	1:13225994	SNV	G>A, C, T	Missense variant
CACNA1A	1233829101	9	19:13208892	SNV	T>G	Missense variant
ARMCX4	1312289720	9	X:101490980	DELINS	T>C, G	Missense variant

Supplementary Table 3. List of driver single genes and gene combinations associated with prognosis in Korean CRC patients.

Gene	Pval	HR	Median (MT)	Mutant	Median (WT)	WT
TP53	0.816	1.12	672	88	721	75
KRAS	0.774	1.15	688	67	698.5	96
HRNR	0.217	1.99	619	20	689	143
ZNF676	0.692	0.667	689	13	686	150
APOL1	0.273	2.24	700.5	10	684	153
PLIN4	0.658	1.29	696.5	28	688	135
LILRB2	0.341	3.83E-08	766	7	686	156
MAP1A	0.876	0.889	613	22	691	141
TBP	0.858	1.2	543	10	689	153
ESRRA	0.68	1.36	684	13	688.5	150
CBWD5	0.0217	3.86	680.5	8	688	155
CCDC73	0.442	2.17	633	4	688	159
CPEB2	0.665	1.11E-07	582.5	2	688	161
CKAP4	0.371	2.44	602	4	689	159
LMTK3	0.412	3.90E-08	755	5	686	158
ZNF595	0.745	3.02E-07	593	1	688.5	162
LILRB3	0.461	3.94E-08	664	6	688	157
DHRS4	0.0596	2.79E+00	647	16	689	147
LRRIQ3	0.0314	4.41E+00	476	5	690	158
TRIM16	0.59	1.09E-07	490	3	688.5	160
TRIM64B	0.0149	5.18E+00	685	4	688	159
UBXN11	0.539	1.08E-07	773	3	686	160
KRTAP10-4	0.917	1.08E+00	632	17	701	146
OR10G2	0.489	3.96E-08	493	5	688.5	158
RUNX1	0.831	8.03E-01	769	10	684	153
TTC33	0.531	1.08E-07	760	3	680	160
NBPF9	0.64	6.19E-01	767.5	12	676	151
TEAD2	0.435	3.91E-08	597	6	689	157
ZNF705G	0.844	1.22E+00	550	8	689	155
ACVR2A	0.855	1.21E+00	731	7	686	156
AGAP9	0.978	1.02E+00	697.5	18	688	145
ANXA8L1	0.529	1.89E+00	761.5	4	684	159
ARMCX4	0.104	3.20E+00	489	8	711	155
ATN1	na	na	na	0	na	163
C1QTNF5	0.495	3.96E-08	635.5	4	689	159
CELA1	0.72	3.02E-07	755	1	686	162

CENPB	0.489	3.96E-08	660	4	688	159
DDX27	0.31	2.74E+00	488	4	689	159
EVX2	0.341	2.58E+00	493	5	690	158
FTSJ3	0.72	3.02E-07	773	1	686	162
GPRIN1	0.627	1.10E-07	683	2	688	161
HCAR2	0.808	1.28E+00	751	7	686	156
HLA-DQB1	0.587	1.09E-07	576	3	688.5	160
KCNJ18	0.847	1.13E+00	777	21	657	142
KRTAP9-6	0.544	1.09E-07	770	3	686	160
LMO2	0.484	3.95E-08	635.5	4	689	159
LRRC37A2	0.513	1.08E-07	588.5	4	689	159
MZF1	0.402	4.32E-01	685	20	688	143
NDUFC2	0.335	2.61E+00	545	4	689	159
NPHP1	0.806	1.29E+00	762.5	6	684	157
NPY4R	0.472	3.94E-08	593	5	688.5	158
NRAS	0.485	2.02E+00	812	4	684	159
OR2T2	0.54	1.86E+00	577	5	688.5	158
OR2T29	0.477	2.05E+00	668	4	688	159
PCBP1	0.454	3.93E-08	653	5	688.5	158
PIK3CA	0.104	1.21E-08	711	19	680	144
POU3F3	0.478	3.95E-08	756	4	684	159
PRRT1	0.475	3.95E-08	768	4	684	159
RBM33	0.536	1.08E-07	524	4	691	159
RBMXL 1	0.188	2.59E+00	755	7	686	156
RPL22	0.725	1.43E+00	686.5	6	688	157
SAMD1	0.238	3.17E+00	461	4	689	159
SLC23A2	0.433	2.20E+00	743	4	684	159
SLC9B1	0.583	1.75E+00	536	6	689	157
SMAD4	0.368	3.85E-08	748.5	6	684	157
SMC6	0.577	1.76E+00	618	5	688.5	158
SPINK5	0.0083	5.80E+00	545	4	689	159
SPRED3	0.394	3.83E-08	822	5	680	158
SRRT	0.433	2.20E+00	760.5	4	684	159
TFAM	0.444	3.92E-08	731	5	686	158
THAP5	0.335	2.61E+00	532.5	4	689	159
TXNDC2	0.585	1.09E-07	412.5	4	691	159
VPS37C	0.714	1.46E+00	776.5	6	676	157
WBP1	0.433	2.20E+00	765	4	684	159
XPOT	0.477	2.05E+00	693.5	4	688	159
ZMI22	0.468	3.94E-08	588	5	689.5	158

ZNF780B	0.513	1.08E-07	569	4	691	159
ZNRF2	0.01	9.18E+00	352.5	2	689	161
Gene combination	**Pval**	**HR**	**Mutant**	**WT**		
APC_PRAMEF10	0.00695	6.01	5	158		
KRAS_TTN	0.0422	2.8	19	144		
TP53_KRAS_APC	0.000121	7.99	7	156		
TP53_KRAS_TTN	0.00771	5.9	5	158		
TP53_KRAS_TTN	0.0156	4.1	8	155		
KRAS_APC_DHRS4	0.0174	5.02	5	158		
TP53_TTN_GOLGA6L9	0.0315	4.39	158	5		

Supplementary Table 4. List of mutations that occurred in hypo mutated MSS.

Gene	dbSNP accession	Frequency	Base substitution	Korean MAF	Clinical significance	Consequence
KRAS	121913529	46	C>A,G,T	Not reported	Not reported	Missense variant
NBPF20	1277995182	21	C>T	T=0.117385	Not reported	Missense variant
MAP1A	771126505	20	T>A,G	A=0.	Not reported	Missense variant
GOLGA6L6	200467851	19	A>C,G	G=0.00999	Not reported	Stop lost
RPTN	200476424	18	G>T	T=0.052361	Not reported	Missense variant
AMH	1286108898	17	A>C	C=0.02141	Not reported	Missense variant
TP53	28934578	15	C>A,G,T	Not reported	pathogenic	Missense variant
TCAF2	761564016	15	T>A,C	C=0.04217	Not reported	Intron variant
HDHD5	1263650512	13	A>C,G,T	Not reported	Not reported	Intron variant
FAM186A	1428760655	13	T>C,G	G=0.08042	Not reported	Missense variant
IFITM3	199749095	13	G>A,C,T	T=0.07598	Not reported	Missense_variant
PLIN4	28523592	13	T>C	Not reported	Not reported	Missense variant
ARHGAP33	1346720614	12	A>C	C=0.059269	Not reported	Missense variant
ZNF676	201994000	12	C>A,G,T	T=0.00308	Not reported	Missense variant
MDC1	748601560	12	C>A,T	A=0.05123	Not reported	Missense variant
ESRRA	759464632	12	GGG>-	Not reported	Not reported	Inframe Indel
APOL1	769299217	12	G>A,T	A=0.016376	Uncertain significance	Missense variant
KRAS	112445441	11	C>A,G,T	Not reported	Likely pathogenic	Missense variant
MUC5AC	1312330802	11	C>A,T	Not reported	Not reported	Missense variant
FRG1FP	1368579899	11	G>A,T	Not reported	Not reported	Synonymous_variant
APOL1	200136783	11	T>C	Not reported	Not reported	Missense variant

	1201123486	11	G>A	Not reported	Not reported	
SLC9B1	201513710	11	A>G	G=0.06438	Not reported	Missense variant
FLG	554101492	11	G>A,C,T	A=0.007529	benign	Missense variant
PABPC3	746078288	11	C>G,T	T=0.024641	Not reported	Missense variant
DHRS4	11556285	10	G>C,T	T=0.01232	Not reported	Missense variant
LOC100287792	1227603601	10	->A,AA,AAA,	Not reported	Not reported	DELINS
TRA2A	1422277440	10	T>A	A=0.07745	Not reported	Splice Acceptor variant
PABPC3	1430736305	10	G>A	A=0.021903	Not reported	Missense variant
MAGEE1	1453916896	10	A>C,G	C=0.01955	Not reported	Missense variant
APOL1	199650512	10	C>T	T=0.016376	Not reported	Missense variant
HNRNPCL2	199999587	10	T>C	C=0.01095	Not reported	Missense variant
FLG	200713352	10	G>A,C	C=0.01061	Not reported	Missense variant
FLG-AS1	201979853	10	C>A,T	A=0.044148	Not reported	Missense variant
SEMG2	771458905	10	C>A	A=0.011294	Not reported	Missense variant
DHRS4	772099993	10	C>A	A=0.003422	Not reported	Missense variant
HNRNPCL2	781034745	10	C>T	T=0.022603	Not reported	Missense variant
NBPF9	9442084	10	G>C,T	Not reported	Not reported	Missense variant
PRAMEF2	1063784	9	A>T	T=0.091376	Not reported	Missense variant
CACNA1A	1176428893	9	T>G	Not reported	Not reported	Missense variant
CACNA1A	1233829101	9	T>G	Not reported	Not reported	Missense variant
TEX13C	1240648466	9	A>C,G	C=0.034565	Not reported	Missense variant
CAPNS1	1244270904	9	T>C,G	G=0.0625	Not reported	Missense variant
ARMCX4	1312289720	9	T>C,G	G=0.00719	Not reported	Missense variant
TRA2A	1320722484	9	C>A	A=0.04586	Not reported	Stop lost
KRT15	1372388443	9	A>C,G	C=0.01748	Not reported	Missense variant

RFPL4A	140087406	9	T>A,G	G=0.09573	Not reported	Missense variant
FLG-AS1	140669858	9	G>A,T	T=0.06708	Not reported	Missense variant
BICRA	1422397217	9	A>C	C=0.07524	Not reported	Missense variant
TCHH	181128140	9	C>A,G,T	A=0.043463	Not reported	Missense variant
TSPEAR	372822453	9	G>A	A=0.000685	Not reported	Missense variant
ADAMTS2	376054177	9	T>G	G=0.062971	Not reported	Missense variant
MYO15B	534573357	9	T>C,G	G=0.01822	Not reported	Synonymous variant
FLG	536240526	9	G>C,T	T=0.010609	Not reported	Stop gained
PABPC3	759092569	9	C>A,T	A=0.066051	Not reported	Synonymous variant
PABPC3	776001979	9	A>G,T	T=0.069815	Not reported	Missense variant
MAGEE1	781934228	9	A>C,G,T	C=0.032214	Not reported	Missense variant
PRAMEF18	878860150	9	G>A,C,T	Not reported	Not reported	Missense variant
TEX13D	1023730611	8	G>A	A=0.03696	Not reported	Missense variant
FAM47C	1190179737	8	A>C,T	C=0.01476	Not reported	Missense variant
VPS37C	1318648573	8	A>C	C=0.00857	Not reported	Missense variant
MIR662	1337385240	8	T>G	G=0.0358	Not reported	500B downstream variant
NBPF26	1349857272	8	A>C,G,T	Not reported	Not reported	Missense variant
PCCA	1411096897	8	A>C	Not reported	Not reported	Intron variant
GOLGA6L6	1412367894	8	T>C	Not reported	Not reported	Missense variant
MIR4477A	1433731755	8	T>A,G	Not reported	Not reported	Non coding transcript variant
PTPN23	1434003227	8	A>C	C=0.025916	Not reported	Missense variant
MZF1	181482460	8	T>A	A=0.01164	Not reported	Intron variant
KYAT3	200907077	8	A>T	T=0.079055	Not reported	Intron variant
KYAT3	200923203	8	A>G,T	G=0.090691	Not reported	Missense variant
KRTAP4-4	201753814	8	T>C,G	G=0.018163	Not reported	Missense variant

	1201971362	8	G>A	Not reported	Not reported	Intron variant
RUNX1	202068364	8	A>C,G	Not reported	Not reported	Missense variant
HLA-A	3173419	8	G>A,C,T	C=0.03964	Not reported	Missense variant
KRTAP4-5	377168597	8	T>C,G	G=0.04318	Not reported	Missense variant
FLG	556324776	8	A>C,G	C=0.019849	Not reported	Missense variant
PABPC3	763263349	8	C>G,T	T=0.098563	Not reported	Missense variant
HRNR	768388262	8	CA>-	-=0.05655	Not reported	Frameshift variant
ZNF676	769830637	8	A>C	C=0.002053	Not reported	Missense variant
PABPC3	770226602	8	A>G,T	G=0.087611	Not reported	Missense variant
CACNA1A	772789381	8	T>C,G	G=0.029882	likely-benign	Missense variant
PABPC3	775997189	8	G>A	A=0.094798	Not reported	Missense variant
SPATC1	782308262	8	A>C,G	C=0.020733	Not reported	Missense variant
MUCL3	879139132	8	T>A,C,G	A=0.06571	Not reported	Missense variant
NBPF26	1159820458	7	T>A,C,G	Not reported	Not reported	Missense variant
LMTK3	1208062349	7	T>G	G=0.01484	Not reported	Missense variant
AGAP9	1283007591	7	C>A,T	Not reported	Not reported	Missense variant
ADGRB1	1293896663	7	A>C,G,T	C=0.094347	Not reported	Missense variant
ARHGEF35	1329484837	7	T>C	C=0.03298	Not reported	Missense variant
AMH	1343170927	7	A>C,G	C=0.012474	Not reported	Missense variant
CACNA1A	1353109044	7	T>G	Not reported	Not reported	Missense variant
GGN	1354730030	7	A>C	C=0.00566	Not reported	2KB upstream variant
IRF2BPL	1372631866	7	T>G	G=0.022774	Not reported	Missense variant
FAM117A	1398365139	7	A>C	C=0.07138	Not reported	Missense variant
FLG	144574658	7	A>C,G	C=0.079055	likely-benign	Missense variant
IL9R	147385831	7	G>A	Not reported	benign	Missense variant

MZF1	1475416986	7	G>C	C=0.007882	Not reported	Missense variant
PRAMEF2	17404799	7	G>A,T	A=0.064682	Not reported	Missense variant
PLIN4	200538852	7	C>A,G,T	G=0.000342	Not reported	Missense variant
TP53	28934574	7	G>A,C,T	Not reported	Not reported	Missense variant
TP53	28934576	7	C>A,G,T	Not reported	Not reported	Missense variant
	751314196	7	TTT>-,T,TT	Not reported	benign	
NACAD	756470140	7	G>T	T=0.089322	Not reported	Missense variant
PABPC3	781717999	7	G>A	A=0.027036	Not reported	Missense variant
TCHH	879174342	7	C>A	A=0.012354	Not reported	Missense variant

MAF: Minor allele frequency
